# A commercial blend of macroalgae and microalgae promotes digestibility, growth performance, and muscle nutritional value of European seabass (*Dicentrarchus labrax* L.) juveniles

**DOI:** 10.3389/fnut.2023.1165343

**Published:** 2023-04-17

**Authors:** Cátia S. C. Mota, Olívia Pinto, Tiago Sá, Mariana Ferreira, Cristina Delerue-Matos, Ana R. J. Cabrita, Agostinho Almeida, Helena Abreu, Joana Silva, António J. M. Fonseca, Luisa M. P. Valente, Margarida R. G. Maia

**Affiliations:** ^1^REQUIMTE, LAQV, ICBAS, Instituto de Ciências Biomédicas Abel Salazar, Universidade do Porto, Porto, Portugal; ^2^REQUIMTE, LAQV, Instituto Superior de Engenharia do Porto, Instituto Politécnico do Porto, Porto, Portugal; ^3^CIIMAR, Centro Interdisciplinar de Investigação Marinha e Ambiental, Terminal de Cruzeiros de Leixões, Matosinhos, Portugal; ^4^ICBAS, Instituto de Ciências Biomédicas Abel Salazar, Universidade do Porto, Porto, Portugal; ^5^REQUIMTE, LAQV, Departamento de Ciências Químicas, Laboratório de Química Aplicada, Faculdade de Farmácia, Universidade do Porto, Porto, Portugal; ^6^ALGAplus-Produção e Comercialização de Algas e seus Derivados, Lda, PCI, Ílhavo, Portugal; ^7^ALLMICROALGAE—Natural Products, Pataias, Portugal

**Keywords:** algae blend, digestibility, growth performance, gut integrity, functional value, microalgae, muscle quality, seaweed

## Abstract

Algae can leverage aquaculture sustainability and improve the nutritional and functional value of fish for human consumption, but may pose challenges to carnivorous fish. This study aimed to evaluate the potential of a commercial blend of macroalgae (*Ulva* sp. and *Gracilaria gracilis*) and microalgae (*Chlorella vulgaris* and *Nannochloropsis oceanica*) in a plant-based diet up to 6% (dry matter basis) on digestibility, gut integrity, nutrient utilization, growth performance, and muscle nutritional value of European seabass juveniles. Fish (11.3 ± 2.70 g) were fed with isoproteic, isolipidic, and isoenergetic diets: (i) a commercial-type plant-based diet with moderate fishmeal (125 g kg^−1^ DM basis) and without algae blend (control diet; Algae0), (ii) the control diet with 2% algae blend (Algae2), (iii) the control diet with 4% algae blend (Algae4), and (iv) the control diet with 6% algae blend (Algae6) for 12 weeks. The digestibility of experimental diets was assessed in a parallel study after 20 days. Results showed that most nutrients and energy apparent digestibility coefficients were promoted by algae blend supplementation, with a concomitant increase in lipid and energy retention efficiencies. Growth performance was significantly promoted by the algae blend, the final body weight of fish fed Algae6 being 70% higher than that of fish fed Algae0 after 12 weeks, reflecting up to 20% higher feed intake of algae-fed fish and the enhanced anterior intestinal absorption area (up to 45%). Whole-body and muscle lipid contents were increased with dietary algae supplementation levels by up to 1.79 and 1.74 folds in Algae 6 compared to Algae0, respectively. Even though the proportion of polyunsaturated fatty acids was reduced, the content of EPA and DHA in the muscle of algae-fed fish increased by nearly 43% compared to Algae0. The skin and filet color of juvenile European seabass were significantly affected by the dietary inclusion of the algae blend, but changes were small in the case of muscle, meeting the preference of consumers. Overall results highlight the beneficial effects of the commercial algae blend (Algaessence^®^) supplementation in plant-based diets for European seabass juveniles, but feeding trials up to commercial-size fish are needed to fully assess its potential.

## 1. Introduction

The quest for healthy and nutritious food is one of the main concerns of the Food and Agriculture Organization (FAO) of the United Nations to address food security and the growing demand for animal protein sources by the world's growing population, expected to reach 9.7 billion by 2050 (1). According to the latest report, severe food insecurity in 2021 increased compared to 2020 (2), mainly due to the long-lasting effects of the COVID-19 pandemic. A trend that may continue as a result of the ongoing war and the political, economic, and financial uncertainty. Indeed, FAO anticipates that 670 million people may suffer from hunger by 2030, a figure similar to 2015 when the Sustainable Development Goals of the 2030 Agenda was launched (2). It is thus of paramount importance to produce high-quality foods to address food insecurity.

Aquaculture, the fastest-growing sector of the food industry, has the potential to contribute to food security and meet the nutritional requirements of the world's growing population (3). To address the growing demand for aquafeeds and the limited supply of fishmeal and fish oil, fewer marine sources and more plant sources are currently used in nutrient-based formulations that not only meet fish nutrient and energy requirements but also attend to their nutraceutical and functional properties (4, 5). These modern formulations of aquafeed pose challenges to fish, in particular to carnivorous species such as European seabass (*Dicentrarchus labrax* L.), a marine species of high commercial importance in European aquaculture, particularly in the Mediterranean region (6). Compared to marine sources, land plant sources have lower digestibility, are deficient in essential amino acids, such as lysine and methionine, and have a lipid profile rich in *n*-6 and completely lacking *n*-3 long-chain polyunsaturated fatty acids (LC-PUFA) (7). The search for alternative, locally produced, and more sustainable ingredients to be included in modern aquafeed formulations is of paramount importance to reduce or replace traditional marine sources, as well as plant ingredients with a high environmental footprint, nutrient imbalance, or antinutritional compounds or that are used for terrestrial animal feeding or human consumption (8). By addressing aquaculture sustainability and improving the nutritional and functional value of fish for human consumption, the search for alternative aquafeed ingredients fits into the One Health concept and the goal to achieve the best health outcomes for fish, consumers, and the environment.

Most research on alternative aquafeed ingredients has focused on alternative plants, rendered animal and aquaculture by-products, insects, single-cell organisms, and algae (8–10). Algae, including microalgae and macroalgae, are of particular interest due to their high growth rates and biomass productivity, low environmental footprint, and non-competition with other cultures for arable land, being even able to grow in waste water (11). In addition, algae are valuable sources of macro- and micro-nutrients and bioactive compounds (12–14), with levels varying with species and within species with abiotic and biotic growth conditions (15, 16). Microalgae contain all essential amino acids, and some species are rich sources of protein and lipids, with marine species being particularly rich in *n*-3 LC-PUFA, such as eicosapentaenoic acid (EPA; C20:5 *n*-3) and docosahexaenoic acid (DHA; C22:6 *n*-3) (17–19) with important health-promoting properties (20). On the contrary, macroalgae species have lower protein content with a less balanced amino acids profile, in particular, brown macroalgae, and are poor sources of lipids, although marine species have a fairly high proportion of LC-PUFA (16, 21). Both microalgae and macroalgae are good sources of complex polysaccharides, pigments, and organic minerals with potential health-promoting effects such as prebiotic, immunomodulatory, and antioxidant activities (12, 14, 22, 23). Although algae lack lignin and are poor in hemicellulose (24, 25), the complex structural polysaccharides of eukaryotic microalgae and macroalgae may reduce the digestibility and availability of macro- and micronutrients for carnivorous fish (16, 18, 26). In face of their nutritional value, the potential of micro- and macroalgae as alternative aquafeeds has been suggested for fingerlings and fish diets, including the replacement of traditional (fishmeal) and current (plant-based) protein sources (10, 18, 25) and lipid sources such as fish oil (27). In addition, algae are sustainable and valuable sources of bioactive compounds, in particular, *n*-3 LC-PUFA, that are essential for aquafeed formulations (12, 28). Although their high production costs are still a bottleneck for algae use in fish diets as ingredients, it is anticipated that in the near future this limitation can be overcome (25).

In this context, several studies have evaluated the inclusion of individual species of microalgae or macroalgae in carnivorous fish diets, namely Atlantic salmon (*Salmo salar*) (29), European seabass (30–32), turbot (*Scophthalmus maximus*) (33), barramundi (*Lates calcarifer*) (34), meager (*Argyrosomus regius*) (35), Persian sturgeon (*Acipenser persicus*) (36), and Senegalese sole (*Solea senegalensis*) (37). In most studies, growth or feed efficiency was not impaired at dietary inclusion levels up to 10% (dry matter, DM, basis), but in some cases, decreased nutrient and energy digestibility was reported (13, 26). At higher inclusion levels, growth performance, feed utilization, and digestibility of diets were reduced (38–41). Available data support species-specific and dose-dependent effects of dietary algae inclusion (42) and highlight the need for further studies.

*Chlorella* spp. and *Nannochloropsis* spp. are among the most produced microalgae species in Europe (82 and 21 tons DM year^−1^, respectively) (43). *Chlorella vulgaris* is a rich source of protein (51.5–67.7% DM basis) (18), with a high content of essential amino acids, particularly arginine, lysine, and leucine (44), while *N. oceanica* is an oleaginous microalga with the ability to accumulate EPA, a health-promoting *n*-3 LC-PUFA (20). Although produced on a small scale at the European level, the production of macroalgae species native to the Mediterranean, such as *Ulva* spp. and *Gracilaria* spp., has a considerable expression in Portugal (43). These macroalgae species are rich sources of polysaccharides (30–75% DM basis) (16), including sulfated polysaccharides, such as ulvan in *Ulva* spp. and carrageenan and agar in *Gracilaria* spp. (45), with prebiotic, immunomodulatory, and antioxidant properties (46–48).

Although the combination of algae species has been suggested to improve the nutritional and functional value of diets (49), few studies have evaluated the supplementation of mixtures of inclusion of microalgae or macroalgae species in European seabass diets (50–53), and only one study has evaluated the combination of one micro- and one macroalgae species in an *in vivo* trial with *D. labrax* (54). We hypothesize that the combination of several species of micro- and macroalgae can exert strong synergistic effects and improve the nutritional and functional value of modern plant source-based aquafeeds. Thus, the present study aimed to evaluate the effects of dietary inclusion of an algae blend composed of two macroalgae (*Ulva* sp. and *Gracilaria gracilis*) and two microalgae (*Chlorella vulgaris* and *Nannochloropsis oceanica*) on digestibility, gut integrity, nutrient utilization, growth performance, and muscle quality of European seabass juveniles.

## 2. Materials and methods

Two trials were conducted at the Fish Culture Experimental Unit of CIIMAR (Matosinhos, Portugal) to evaluate the potential of the algae blend as a novel feed for European seabass juveniles: a digestibility trial and a growth trial. All procedures with animals were reviewed and approved by the Animal Welfare and Ethics Body of CIIMAR (ORBEA-CIIMAR 06-2016), licenced by the Portuguese Veterynary Authority (1005/92, DGAV-Portugal), and carried out by trained researchers accredited in laboratory animal science following FELASA C recommendations. The experiments were conducted in strict compliance with European Union guidelines on the protection of animals for scientific purposes (Directive 2010/63/EU).

### 2.1. Fish

European seabass (*D. labrax*) juveniles were obtained from a commercial fish farm (Acuinuga, S.L., La Coruña, Spain), transported to CIIMAR facilities, and kept in quarantine for 3 weeks. During this period, the fish were fed a commercial diet (49% crude protein, CP, and 20% ether extract, EE, DM basis; AQUASOJA, Sorgal, S.A., Ovar, Portugal). After acclimation, the fish were fasted for 24 h, anesthetized (60 μl L^−1^ of 2-phenoxyethanol, Sigma-Aldrich, St. Louis, MO, USA), and individually weighted (g) and measured (total length, cm). Homogeneous groups of fish were then distributed among the tanks used for the digestibility and growth trials.

### 2.2. Algae blend and experimental diets

The algae blend used in this study, composed of two macroalgae species (*Ulva* sp. and *G. gracilis*) and two microalgae species (*C. vulgaris* and *N. oceanica*), is a commercial product (Algaessence^®^ feed) produced by ALGAplus (Ílhavo, Portugal) and Allmicroalgae (Pataias, Portugal). The blend was supplied as a spray-dried power in sealed bags protected from light.

Four isoproteic (527 g kg^−1^ DM basis), isolipidic (153 g kg^−1^ DM basis), and isoenergetic (21.7 MJ kg^−1^ DM basis) diets were formulated according to the nutritional requirements of European seabass juveniles (55) and considering current trends in commercial aquafeeds of high vegetable protein sources (c.a., 700 g kg^−1^ DM basis) and moderate fishmeal (125 g kg^−1^ DM basis) inclusion level. The algae blend was included in the experimental diets at expense of wheat gluten and whole peas, with levels of fishmeal and fish oil held constant. The experimental diets were as follows: i) a commercial plant protein-based diet without algae blend inclusion (control diet; Algae0), ii) the control diet with 2% algae blend inclusion (Algae2), iii) the control diet with 4% algae blend inclusion (Algae4), and iv) the control diet with 6% algae blend inclusion (Algae6). Yttrium oxide (Y_2_O_3_, 0.2 g kg^−1^, DM basis) was included in all diets as an inert marker for determining the apparent digestibility coefficients. Diets were manufactured and extruded by SPAROS Lda. (Olhão, Portugal), using a pilot-scale twin-screw extruder (CLEXTRAL BC45, Firminy, France). The pellets (2.0 mm) were dried in a convection oven (OP 750-UF, LTE Scientific, Oldham, UK), and the fish oil was added by vacuum coating (Pegasus PG-10VCLAB, DINNISSEN, Sevenum, Netherlands). The diets were stored at 4°C until use. The ingredients and chemical composition of the experimental diets are shown in Table 1 and Supplementary Table S1.

**Table 1:** Ingredient composition (g kg^−1^, as is) and proximate composition, essential amino acids, and selected fatty acids content (g kg^−1^, dry matter) of the algae blend and experimental diets.

	**Algae blend**	**Diets**			
		**Algae0**	**Algae2**	**Algae4**	**Algae6**
**Ingredient composition**					
Norwegian fishmeal LT 70^a^		125	125	125	125
Soy protein concentrate^b^		300	300	300	300
Wheat gluten^c^		110	105	101	96.0
Corn gluten^d^		125	125	125	125
Soybean meal 48^e^		100	100	100	100
Whole peas		82.8	68.8	52.8	37.8
Fish oil^f^		132	131	131	131
Vitamin and mineral premix^g^		5.00	5.00	5.00	5.00
Monocalcium phosphate^h^		20.0	20.0	20.0	20.0
Yttrium oxide		0.200	0.200	0.200	0.200
Algae blend^i^		-	20.0	40.0	60.0
**Proximate composition**					
Ash	251	74.2	78.6	83.0	86.4
Crude protein	347	526	529	525	530
Total lipids	59.5	128	130	121	129
Crude fiber	24.5	29.0	28.1	27.1	25.3
Neutral detergent fiber	126	120	131	127	121
Acid detergent fiber	53.2	60.4	60.4	61.6	62.0
Starch	21.7	55.7	50.6	45.8	38.7
Gross energy (MJ kg^−1^)	16.5	21.8	21.5	21.7	21.8
Essential amino acids	187	261	254	249	250
Arginine	28.7	41.7	40.5	38.9	39.5
Histidine	6.08	13.3	12.4	11.8	11.8
Isoleucine	15.9	23.4	23.1	22.8	23.3
Leucine	27.3	41.0	41.6	40.8	40.7
Lysine	30.2	29.0	31.3	31.4	33.1
Methionine	7.02	10.2	8.87	9.02	8.91
Methionine + Cystine	8.62	13.4	12.0	11.7	11.5
Phenylalanine	18.9	29.4	26.4	26.1	24.9
Phenylalanine + Tyrosine	35.4	54.3	49.7	48.9	46.2
Threonine	15.4	21.1	20.7	20.4	20.3
Valine	19.1	24.1	22.9	23.0	23.2
Fatty acids	54.8	159	161	162	162
C16:0	13.5	31.6	31.5	31.4	31.2
C18:0	0.536	6.13	6.05	6.16	6.08
C16:1 *n*-7	7.39	12.4	12.5	12.5	12.5
C18:1 *n*-9	2.13	17.9	17.7	17.5	17.2
C18:2 *n*-6	4.00	10.7	10.6	10.3	10.1
C18:3 *n*-3	5.52	1.81	1.92	2.00	2.09
C20:5 *n*-3 (EPA)	5.93	21.1	22.3	23.1	23.6
C22:6 *n*-3 (DHA)	ND	15.3	15.2	15.3	15.2
EPA + DHA	5.93	36.4	37.5	38.4	38.8

Algae0, commercial-based diet without algae blend inclusion (control diet); Algae2, control diet with 2% algae blend inclusion; Algae4, control diet with 4% algae blend inclusion; Algae6, control diet with 6% algae blend inclusion; EPA, eicosapentaenoic acid; DHA, docosahexaenoic acid; ND, not detected.

a NORVIK LT 70: 70.6% crude protein (CP), 5.8% ether extract (EE) (Sopropêche, Wimille, France).

b Soycomil^®^-P: 63% CP, 0.8% EE (ADM, Amsterdam, The Netherlands).

c Wheat gluten meal: 10.2% CP, 1.2% EE (Casa Lanchinha, Alhos Vedros, Portugal).

d Corn gluten meal: 61% CP, 6% EE (COPAM, São João da Talha, Portugal).

e Solvent extracted dehulled soybean meal: 47% CP, 1.6% EE (CARGILL, Barcelona, Spain).

f Fish oil (Sopropêche, Wimille, France).

g Vitamins (IU or mg/kg diet): 100 mg DL-alpha tocopherol acetate, 25 mg sodium menadione bisulfate, 20,000 IU retinyl acetate, 2.000 IU DL-cholecalciferol, 30 mg thiamin, 30 mg riboflavin, 20 mg pyridoxine, 0.1 mg cyanocobalamin, 200 mg nicotinic acid, 15 mg folic acid, 500 mg ascorbic acid; 500 mg inositol, 3 mg biotin, 100 mg calcium pantothenate, 1,000 mg choline chloride, 500 mg betaine; Minerals (g or mg/kg diet): 9 mg copper sulfate, 6 mg ferric sulfate, 0.5 mg potassium iodide, 9.6 mg manganese oxide, 0.01 mg sodium selenite, 7.5 mg zinc sulfate, 400 mg sodium chloride; excipient: wheat middling's (Wisium, Cantanhede, Portugal).

h Monocalcium phosphate: 21.8% P, 18.4% Ca (Fosfitalia, Ravenna, Italy).

i Algaessence^®^ feed (Allmicroalgae/ALGAplus, Portugal).

#### 2.2.1. Proximate analysis

The algae blend and the ground (1-mm) experimental diets were homogenized, and their proximate composition was analyzed in duplicate according to official methods (56). Samples were analyzed for DM (ID 934.01), ash (ID 942.05), and nitrogen (N) (ID 990.03) contents. Crude protein was calculated as N x 6.25 (ID 990.03). Gross energy (GE) was determined using an adiabatic bomb calorimeter (Werke C2000, IKA, Staufen, Germany). The starch content was analyzed in 0.5-mm ground samples (57). Crude fiber (CF; ID 962.09), neutral detergent fiber (NDF), and acid detergent fiber (ADF) (58, 59) of the algae blend and diets were also determined. Due to the small size of the microalgae species present in the blend (< 25 μm diameter), the filtration step to determine the fiber content (CF, NDF, and ADF) was modified by replacing the P2 crucibles (porosity 40–100 μm) with glass microfiber filter (Whatman GF/A, 1.6 μm porosity, Merck KGaA, Darmstadt, Germany). Fiber fractions were expressed exclusive of residual ash. Analyses were run in duplicate.

#### 2.2.2. Lipids and fatty acids analyses

The total lipids were quantified following the method of Folch et al. (60), modified by using dichloromethane:methanol (2:1) instead of trichloromethane:methanol (2:1). The fatty acids were transesterified to fatty acid methyl esters by acid-catalyzed methylation (61). Non-adecanoic acid (C19:0, Matreya LLC, State College, PA, USA) was added as an internal standard. Fatty acid methyl esters were analyzed by gas chromatography, using a Shimadzu GC-2010 Plus gas chromatograph (Shimadzu Europe GmbH, Duisburg, Germany) equipped with a capillary column (Omegawax 250, 30 m × 0.25 mm × 0.25 μm; Supelco, Bellefonte, PA, USA) and a flame-ionization detector. The carrier gas was helium at 1.30 ml min^−1^, with a split ratio of 1:100, and the injection volume was 1.0 μl. The initial column temperature of 150°C was held for 7 min, increased at 3°C min^−1^ to 170°C and held for 25 min, and then increased at 3°C min^−1^ to 220°C and held for 30 min. The injector and detector temperatures were 250 and 260°C, respectively. Fatty acids were identified by comparing retention times with those of commercially available standards (Supelco 37 Component FAME Mix, BAME Mix, PUFA No.1, PUFA No.2, PUFA No.3, Sigma-Aldrich Co. LLC; GLC-110 Mixture, Matreya LLC) and quantified by using the internal standard (C19:0). Analyses were run in duplicate.

#### 2.2.3. Amino acids analysis

The amino acid content of the algae blend and experimental diets were determined after hydrolysis with 6 M HCl at 116°C, for 48 h, followed by pre-column derivatization with 6-aminoquinolyl-N-hydroxysuccinimidyl carbamate (Waters AccQ Fluor Reagent; Waters, Milford, MA, USA) as described by Aragão et al. (62), using norvaline (Waters) as an internal standard. Amino acids were analyzed by ultra-high-performance liquid chromatography on a Waters reverse phase amino acid analysis system and identified by comparison of retention times of commercial standard mixtures (Waters) and pure standards (Sigma-Aldrich Co. LLC). Data were acquired and analyzed using the EMPOWER software (Waters). The analysis was run in duplicate.

#### 2.2.4. Element analysis

Macro and trace elements of algae blend and diets were determined after mineralization in a Milestone (Sorisole, Italy) MLS 1200 Mega high-performance microwave digestion unit (63). Samples were analyzed by inductively coupled plasma-mass spectrometry (ICP-MS; Thermo Fisher Scientific iCAP Q ICP-MS instrument, Waltham, MA, USA) and flame atomic absorption spectrometry (FAAS; PerkinElmer AAnalyst 200 FAAS instrument, Waltham). The calibration standards for FAAS were prepared from single-element standard stock solutions (Fluka, Buchs, Switzerland) by appropriate dilution with HNO_3_ 0.2% (v/v). For ICP-MS determinations, internal standards and tuning solutions were prepared by appropriate dilution of the following solutions: periodic table mix 3 for ICP-MS (TraceCERT^®^, Sigma-Aldrich) containing 10 mg L^−1^ of 16 elements (Sc, Y, La, Ce, Pr, Nd, Sm, Eu, Gd, Tb, Dy, Ho, Er, Tm, Yb, and Lu in 5% HNO_3_) and a custom solution (SCP Science, Quebec, Canada) with 1 mg L^−1^ of Ba, Bi, Ce, Co, In, Li, and U in a 5% HNO_3_ + 0.5% HCl, respectively. The iodine content of the algae blend and diets were also determined. After alkaline extraction with tetramethylammonium hydroxide (TMAH, Sigma-Aldrich Co. LLC) at a high temperature (90 ± 3 °C) for 3 h, iodine was analyzed by ICP-MS, and the concentration was calculated based on external calibration with iodine standards prepared in 0.5% (v/v) TMAH (64). The analyses were carried out in triplicate.

### 2.3. Digestibility trial

From an initial lot of European seabass juveniles, six homogeneous groups of 30 fish (14.1 ± 6.36 g of body weight, BW) were randomly allocated to 50 L fiberglass tanks with individual feces sedimentation columns, in a Guelph system as described by Cho and Slinger (65). Fish were adapted to the new conditions (21.5 ± 0.27°C of water temperature, 35.7 ± 1.15‰ of salinity, 2 L min^−1^ of flow rate, and 12 h of light/dark photoperiod) for 19 days. During this period, fish were fed a commercial diet (49% CP and 20% EE, DM basis; AQUASOJA, Sorgal, S.A.). After acclimatization, the digestibility of the experimental diets was assessed in two runs of 20 days; 7 days for adaptation to each experimental diet and 13 days for total feces collection. The experimental diets were hand-fed until apparent satiation two times a day (9:00 and 17:00 h), 7 days a week. During the feces collection period, the tank and the sedimentation column were thoroughly cleaned after 30 min of feeding to ensure that all uneaten feed was removed. Feces were collected from the sedimentation column two times a day, before feeding, centrifuged at 3,000 x *g* for 5 min at 4°C to eliminate the excess water, and stored at −20°C until further analysis. At the end of the first run, the fish were fasted for 24 h for gut evacuation. After this period, the second run of the digestibility trial began. Diets were randomly distributed by the tanks in the first run and caution was taken to ensure that diets were not allocated to the same tank in the second run. By the end of the digestibility trial, all experimental diets were run in triplicate.

Before further analysis, feces were pooled per tank (*n* = 3), freeze-dried, and sieved. Feces samples were homogenized and analyzed for DM, ash, N (CP calculated as N x 6.25), starch, GE, total lipids, fatty acids, and minerals content according to the methodologies described for the algae blend and experimental diets.

### 2.4. Growth trial

From the initial lot, 12 groups of 46 fish (11.3 ± 2.70 g of BW and 10.5 ± 1.04 cm total length) were randomly allocated to 160 L fiberglass tanks within a saltwater recirculation system. The fish were adapted to the new conditions (20.9 ± 0.43°C of water temperature, 35.8 ± 1.29‰ of salinity, 10 L min^−1^ of flow rate, and 12 h of light/dark photoperiod) for 20 days. After acclimation, the experimental diets were randomly assigned to triplicate groups of fish, which were fed by automatic feeders until apparent satiation three times a day, at 9:00, 13:00, and 17:00 h, 7 days a week. The amount of feed supplied to each tank was adjusted according to the presence or absence of feed in the tank (66). Water quality parameters (pH, nitrogenous compounds, and dissolved O_2_) were monitored daily and maintained within recommended levels for marine fish species (67). The growth trial lasted 12 weeks. Fish were bulk weighed after 5 weeks to monitor weight gain and feed consumption.

Before starting the trial, six fish from the initial lot were sacrificed by anesthetic overdose (1.5 ml L^−1^ of 2-phenoxyethanol, Sigma-Aldrich) and stored at −20°C until whole-body composition analysis. At the end of the growth trial (12 weeks), all fish were fasted for 24 h, anesthetized (60 μl L^−1^ of 2-phenoxyethanol, Sigma-Aldrich), and individually weighted (g) and measured (total length, cm). Six fish per tank were sacrificed by anesthetic overdose (1.5 ml L^−1^ of 2-phenoxyethanol, Sigma-Aldrich) for further whole-body composition analysis, and the remaining fish were sacrificed by a sharp blow to the head. The viscera and liver of 12 fish per tank were weighted (g) to determine the viscerosomatic and hepatosomatic indices. Six fish per tank were collected for skin, muscle, and intestine analysis. Left dorsal skin and muscle were collected and color was immediately assessed. Then, the instrumental texture of the muscle was determined. The right dorsal muscle was collected, snap frozen, and kept at −80°C until nutritional value analysis (DM, CP, total lipids, and fatty acids profile). A section of ~0.5 cm of the anterior intestine (after the pyloric ceca) was collected, rinsed, and fixed in a 10% neutral-buffered formalin for 24 h, and then transferred to 70% ethanol until histomorphological evaluation.

### 2.5. Intestine histomorphology evaluation

Fixed samples of the anterior intestine from three fish per tank (nine fish per diet) were selected for histological analysis and embedded in paraffin. The embedded tissues were cut into 3 μm sections by a semiautomated rotary microtome (Leica RM 2245, Leica Biosystems, Nussloch, Germany). For quantitative analysis, sections were stained with specific Alcian blue/PAS (pH 2.5) and observed under a light microscope (Olympus BX51; Olympus, Tokyo, Japan) with a camera (Olympus DP50; Olympus). In each section of the samples, cross-sectional perimeter (mm), muscularis externa thickness (μm), submucosa width (μm), lamina propria width (μm), absorption area (mm^2^), and villus length and width (μm) were measured, and neutral (magenta) and acid (blue) goblet cells were counted using imaging software (Olympus cellSens Dimension Desktop; Olympus). For muscularis externa thickness and submucosa and lamina propria width, eight points of each section were measured, and the average value was calculated. Fold's length was measured in the eight highest folds from the folding tip to the bottom, following the curves of the fold. Goblet cell counts were expressed per villus area.

Intestinal integrity was evaluated using a semi-quantitative analysis: cross-sectional intestinal sections were stained with hematoxylin/eosin and a range of tissue scores set from 1 (normal tissue) to 5 (highly altered) of submucosa and lamina propria cellularity, mucosal folds, inflammatory infiltrates, and enterocytes nucleolus position (68, 69).

### 2.6. Whole-body composition

Before analysis, fish collected for whole-body composition were pooled per tank (*n* = 12), minced frozen in a commercial meat grinder, and freeze-dried. Whole-fish samples were homogenized and analyzed for DM [ID 934.01, (56)], ash [ID 942.05, (56)], EE [ID 920.39, (56)], and N [ID 990.03, (56)] contents. Crude protein was calculated as N x 6.25, and GE content was determined using an adiabatic bomb calorimeter. All analyses were run in duplicate.

### 2.7. Muscle nutritional value

Dorsal muscle samples were freeze-dried and pooled per two individuals per tank (*n* = 9). Muscle samples were homogenized and analyzed for DM and N contents (56), with CP being calculated as N x 6.25. Total lipids of muscle samples were extracted with dichloromethane:methanol (2:1) and determined gravimetrically (60). Fatty acids methyl esters were prepared by direct acid-catalyzed transmethylation (61) and analyzed by gas chromatography as described for the algae blend and experimental diets. The analyses were performed in duplicate.

### 2.8. Skin and muscle color

Skin and muscle color was assessed using a CR-400 Chroma Meter (Konica Minolta Inc., Osaka, Japan) with an aperture of 8 mm, at the CIE D65 standard illuminant. The color was expressed in CIELAB coordinates, where L^*^ measures the degree of lightness (on a scale of 0 to 100, from black to white), a^*^ the degree of redness/greenness (+ red and – green), and b^*^ the degree of yellowness/blueness (+ yellow and – blue). The colorimeter was calibrated against a white plate reference standard (L^*^ = 98.0; a^*^ = 0.3; b^*^ = 2.4; Minolta Co. Ltd.). Color measurements were made by leaning the colorimeter on the surface of the skin and muscle, at three points per fish (54, 66). After flashing, the reflected light values were saved, and the Hue angle (h^*^ = tan^−1^ b^*^ / a^*^) and Chroma (C^*^ = (a^*2^ + b^*2^)^1/2^) values were calculated.

### 2.9. Muscle texture

Instrumental dorsal muscle texture was determined using a TA.XT Plus Texture Analyzer equipped with a 2.0-mm diameter probe (Stable Micro Systems, Surrey, UK). The texture profile was obtained in a sequence of two compressions, with a 5-kg load cell, a constant speed of 1.0 mm s^−1^, and a penetration depth of 4.0 mm. Compressions were made 5 s apart at three points of the thickest part of each filet. Texture data were analyzed using Exponent v6 software (Stable Micro Systems), and the texture parameters hardness (N), adhesiveness (J), chewiness (J), springiness, cohesiveness, and resilience were determined (54, 66).

### 2.10. Calculations

Growth performance parameters were calculated based on BW and body length, as follows:


$$\begin{array}{l} Average\;body\;weight\;\left(ABW\right)=\frac{final\;BW+initial\;BW}{2}\;\\ Daily\;growth\;index\;\left(DGI\right)=\left(\frac{{final\;BW}^{\frac{1}{3}}-\;{initial\;BW}^{\frac{1}{3}}}{days}\right)100\;\\ Specific\;growth\;rate\;\left(SGR\right)=\left(\frac{final\;BW-\;initial\;BW}{days}\right)100\;\\ Condition\;factor\;\left(K\right)=\left(\frac{final\;BW}{{final\;body\;length}^{3}}\right)100\;\\ Hepatosomatic\;index\;\left(HSI\right)=\left(\frac{liver\;weight}{BW}\right)100\;\\ Viscerosomatic\;index\;\left(VSI\right)=\left(\frac{viscera\;weight}{BW}\right)100\; \end{array}$$

Feed efficiency parameters were calculated based on feed intake corrected for the number of fish lost due to mortality and/or sampling, as follows:


$$\begin{array}{l} Voluntary\;feed\;intake\;\left(VFI\right)=\frac{dry\;feed\;intake}{ABW\;days}\;\\ Feed\;conversion\;ratio\;\left(FCR\right)=\frac{dry\;feed\;intake}{weight\;gain}\;\\ Protein\;efficiency\;ratio\;\left(PER\right)=\frac{weight\;gain}{CP\;intake}\; \end{array}$$

The apparent digestibility coefficients (ADC) of the experimental diets were calculated based on the amount of yttrium oxide in diets and feces as proposed by Maynard et al. (70):

The nutritional quality indices of lipids in juvenile European seabass filets were calculated according to Chen and Liu (71) as follows:


$$\begin{array}{lll} Dry\;matter\;ADC\;\left(\%\right) & = & 100\left(1-\left(\frac{dietary\;Y_{2}O_{3}}{fecal\;Y_{2}O_{3}}\right)\right)\;\\ Nutrient\;or\;energy\;ADC\;\left(\%\right) & = & 100\left(1-\left(\frac{dietary\;Y_{2}O_{3}}{fecal\;Y_{2}O_{3}}\right)\left(\frac{fecal\;nutrient\;or\;energy}{dietary\;nutrient\;or\;energy}\right)\right)\;\\ Nutrient\;or\;energy\;gain & = & \frac{final\;carcass\;nutrient\;or\;energy-initial\;carcass\;nutrient\;or\;energy}{ABW\;days}\;\\ Digestible\;nutrient\;or\;energy\;intake & = & \frac{\left(dry\;feed\;or\;energy\;intake\right)\;\left(nutrient\;or\;energy\;ADC\right)}{ABW\;days}\;\\ Nutrient\;or\;energy\;retention\;efficiency & = & \left(\frac{nutrient\;or\;energy\;gain}{digestible\;nutrient\;or\;energy\;intake}\right)100\;\\ Fecal\;nutrient\;or\;energy\;losses & = & crude\;nutrient\;or\;energy\;intake\;\left(1-\left(\frac{nutrient\;or\;energy\;ADC}{100}\right)\;\right)\;\\ Nonfecal\;nutrient\;losses & = & crude\;nutrient\;intake-nutrient\;gain-fecal\;nutrient\;losses\;\\ Nonfecal\;energy\;losses & = & nonfecal\;nitrogen\;losses\;25\;kJ\;g^{-1}\;\\ Metabolisable\;energy\;\left(ME\right) & = & digestible\;energy\;intake-nonfecal\;energy\;losses\;\\ Total\;heat\;loss & = & ME-energy\;gain\; \end{array}$$

$$\begin{array}{l} Thrombogenicity\;index\;\left(TI\right)\\ =\left(\frac{C14:0+C16:0+C18:0}{0.5\;MUFA+0.5\;PUFA\;n-6+3\;PUFA\;n-3+PUFA\;n-3\;/\;PUFA\;n-6}\right)\;\\ Atherogenicity\;index\;\left(AI\right)=\left(\frac{C12:0+4\;C14:0+C16:0}{PUFA\;n-3+\;PUFA\;n-6+MUFA}\right)\;\\ Hypocholesterolemic\;to\;hypercholesterolemic\;ratio\;\left(h/H\right)\\ =\left(\frac{C18:1\;n-9+C18:2\;n-6+C20:4\;n-6+C18:3\;n-3+C20:5\;n-3+C22:6\;n-3}{C14:0+C16:0}\right)\;\\ Flesh\;lipid\;quality\;\left(FLQ\right)=\;\left(\frac{C20:5\;n-3\;+\;C22:6\;n-3}{total\;fatty\;acids}\right)\;x\;100\; \end{array}$$

### 2.11. Statistical analysis

Data were analyzed using the general linear model (GLM) procedure of SPSS (2009; IBM SPSS statistics V26; IBM, Armonk, NY, USA). The model included the fixed effect of diet (Algae0, Algae2, Algae4, and Algae6) and the random residual error. When statistical differences were observed, multiple comparisons of means were performed using the *post hoc* HSD Tukey test. Effects were considered significant when *p* < 0.05 and a trend when 0.05 ≤ *p* ≤ 0.10.

## 3. Results

### 3.1. Algae blend and experimental diets

The algae blend had moderate CP (347 g kg^−1^ DM) and NDF (126 g kg^−1^ DM) contents, low starch (21.7 g kg^−1^ DM) and GE (16.5 MJ kg^−1^ DM) contents, and high ash content (251 g kg^−1^ DM; Table 1). All amino acids considered essential for European seabass were found in the algae blend (Table 1). Lysine was the most abundant essential amino acid (30.2 g kg^−1^ DM), followed by arginine and leucine (28.7 and 27.3 g kg^−1^ DM, respectively), while methionine and histidine (7.02 and 6.08 g kg^−1^ DM, respectively) were the least abundant. The fatty acids profile of the algae blend was highly unsaturated (Table 1), comprising 37.9% PUFA and 26.8% MUFA. However, the blend had only moderate content of the essential highly unsaturated fatty acid EPA (5.93 g kg^−1^ DM) and no DHA was detected. The algae blend proved to be a good source of macro and trace elements, particularly magnesium, potassium, sodium, aluminum, boron, chromium, copper, iodine, iron, manganese, strontium, and zinc (Supplementary Table S1).

Although experimental diets were formulated to be isoproteic, isolipidic, and isoenergetic, the essential amino acids content was slightly decreased by algae blend inclusion, reflecting its lower content of arginine, histidine, methionine, phenylalanine, and threonine (Table 1). Conversely, the lysine content increased with the inclusion levels of the algae blend. The fatty acids content was globally similar among diets with algae blend inclusion leading to a small decrease in MUFA and an increase in PUFA content (Table 1). The content of magnesium, phosphorus, potassium, sodium, aluminum, cobalt, iron, lithium, manganese, and zinc increased in the diets with algae blend inclusion, while the content of cadmium, chromium, copper, molybdenum, nickel, and selenium decreased (Supplementary Table S1).

### 3.2. Digestibility

The ADC of the nutrients and energy of the experimental diets were greatly affected by algae blend supplementation (*p* < 0.05; Table 2). ADC of DM was 12% higher in Algae4 and Algae6 (72.2 and 73.0%) compared to Algae0 (64.8%); Algae2 did not differ from other diets. Likewise, the algae blend inclusion promoted the ADC of CP and GE, the highest values being found with Algae4 and Algae6 and the lowest with Algae0; Algae2 was similar to other diets. Organic matter and total lipids ADC increased by 9% and 8%, respectively, with algae blend supplementation compared to control (0%), with no differences being observed among inclusion levels.

**Table 2:** Apparent digestibility coefficients (%) of nutrients, energy, and minerals of the experimental diets fed to European seabass juveniles.

	**Diet**				SEM	***p-*** **value**
	**Algae0**	**Algae2**	**Algae4**	**Algae6**		
Dry matter	64.8^a^	68.8^ab^	72.2^b^	73.0^b^	1.13	0.003
Organic matter	74.9^a^	78.3^b^	80.8^b^	81.4^b^	0.75	0.001
Crude protein	91.5^a^	92.5^ab^	93.5^b^	93.4^b^	0.23	< 0.001
Total lipids	82.8^a^	87.7^b^	89.3^b^	89.7^b^	0.99	0.004
Gross energy	79.8^a^	83.2^ab^	85.0^b^	85.6^b^	0.76	0.003
**Fatty acids**						
SFA	72.7^a^	82.8^b^	88.2^b^	88.4^b^	1.91	0.001
MUFA	85.0^a^	91.3^b^	94.3^b^	94.3^b^	1.17	0.001
PUFA	94.6^a^	95.2^ab^	96.5^b^	96.4^b^	0.30	0.006
PUFA *n*-3	95.6^a^	96.2^ab^	97.1^b^	97.0^b^	0.31	0.025
PUFA *n*-6	91.0^a^	91.1^a^	93.7^b^	93.5^b^	0.37	< 0.001
**Elements**						
Calcium	86.3	85.9	86.8	87.2	0.38	0.186
Magnesium	59.7^a^	72.0^b^	75.9^b^	79.1^b^	1.91	< 0.001
Potassium	95.8	96.5	96.5	96.8	0.30	0.236
Phosphorus	64.0	64.1	61.8	61.9	1.22	0.418
Manganese	71.6^ab^	69.6^ab^	67.0^a^	72.7^b^	1.10	0.029
Iron	14.2^c^	12.1^bc^	10.8^b^	4.00^a^	0.556	< 0.001
Copper	81.2^b^	76.4^a^	78.3^ab^	77.2^a^	0.55	0.006
Selenium	14.7	15.6	14.1	15.9	1.36	0.785

Algae0, commercial-based diet without algae blend inclusion (control diet); Algae2, control diet with 2% algae blend inclusion; Algae4, control diet with 4% algae blend inclusion; Algae6, control diet with 6% algae blend inclusion; SEM, standard error of the mean; SFA, saturated fatty acids; MUFA, monounsaturated fatty acids; PUFA, polyunsaturated fatty acids.

a−c Means in the same line with different superscripts are statistically different (*p* < 0.05).

The ADC of most individual fatty acids was affected by dietary algae blend inclusion (*p* < 0.05; Supplementary Table S2), except C10:0, C17:1 *n*-7, C20:3 *n*-6, and C22:4 *n*-6 (*p* > 0.05). In general, the ADC of individual even-chain fatty acids (ECFA), odd-chain fatty acids (OCFA), branched-chain fatty acids (BCFA), and MUFA were promoted (*p* < 0.05) in diets with algae supplementation, with no differences among inclusion levels. The exceptions were C22:0, which was the highest with Algae6, and C13:0 and C20:1 *n*-11, which were the highest with Algae4 and Algae6 compared to Algae0. Effects on individual PUFA ADC were also marked (*p* < 0.05) but less consistent. ADC of C18:2 *n*-6 and C20:2 *n*-6 were the highest with Algae4 and Algae6 compared to Algae0 and Algae2, and ADC of C18:3 *n*-3 was the lowest and the highest with 2% and 4% algae inclusion, respectively. The ADC of C18:3 *n*-6 was the lowest with Algae0 and the highest with Algae4, while C20:4 *n*-6 was the lowest with Algae6 and the highest with Algae0; Algae2 and Algae4 not differing from the other levels. C18:4 *n*-3, C20:3 *n*-3, C20:4 *n*-3, C21:5 *n*-3, and DHA ADC were the highest with Algae4 and Algae6 compared to the control; Algae2 was similar to other levels. In addition, algae blend dietary inclusion also increased the ADC of total ECFA by 19%, OCFA by 20%, BCFA by 15%, total SFA by 19%, and MUFA by 10% compared to Algae0 (*p* < 0.05), with no differences being observed among inclusion levels. Algae4 and Algae6 improved the ADC of total PUFA *n*-3 by 1% and total PUFA by 2% compared to Algae0, and of PUFA *n*-6 by 2% compared to Algae0 and Algae2 (*p* < 0.05).

The ADC of calcium, potassium, phosphorus, zinc, and selenium were not affected by the inclusion of the algae blend (*p* >0.05; Table 2). Magnesium ADC was increased by 27% with algae blend supplementation compared to Algae0, with no differences being observed among inclusion levels. The manganese ADC was the lowest with Algae4 and the highest with Algae6; Algae0 and Algae2 not differing from the other diets. Copper ADC was reduced with algae supplementation; the lowest values were found with Algae2 and Algae6 and the highest with Algae0; Algae4 not differing from the other diets. Similarly, iron ADC decreased with increasing levels of the algae blend, with Algae6 being 72% lower than Algae0 (Table 2).

The effects of algae blend inclusion on N, lipid, and energy balances of European seabass juveniles are shown in Table 3. Digestible N, lipid, energy intake, and lipid gain gradually increased with algae blend inclusion (*p* < 0.05). Nitrogen and energy gains were promoted by the algae blend (*p* < 0.05), but no differences were observed among inclusion levels. Nitrogen retention efficiency was not affected by algae supplementation, while lipid and energy retention efficiencies were increased (*p* < 0.05), regardless of the inclusion level. Fecal N losses were the lowest with Algae4 and the highest with Algae0, while non-fecal N losses increased with the algae blend inclusion levels (*p* < 0.05). Fecal and non-fecal lipid losses were promoted by algae blend supplementation (*p* < 0.05), with no differences being observed among inclusion levels. Non-fecal energy losses were the highest with Algae6 compared to Algae0 (*p* < 0.05). Metabolizable energy gradually increased with algae blend supplementation levels (*p* < 0.05), with Algae6 being 30.7% higher than Algae0. Total heat loss was not affected by the dietary algae blend (*p* > 0.05).

**Table 3:** Nutrient and energy balances of European seabass juveniles fed the experimental diets.

	**Diet**				**SEM**	***p-*** **value**
	**Algae0**	**Algae2**	**Algae4**	**Algae6**		
**Nitrogen (N) balance (mg 100 g ABW**^**−1**^ **day**^**−1**^**)**						
Digestible N (DN) intake	115^a^	130^b^	132^bc^	142^c^	2.7	<0.001
N gain	33.6^a^	40.0^b^	41.8^b^	43.2^b^	1.20	<0.001
N retention efficiency (% DN)	29.3	30.7	31.7	30.4	0.65	0.147
Fecal N losses	10.6^b^	10.5^ab^	9.13^a^	10.1^ab^	0.30	0.036
Non-fecal N losses	81.0^a^	90.4^bc^	89.8^ab^	99.2^c^	2.00	0.002
**Lipid (L) balance (mg 100 g ABW**^**−1**^ **day**^**−1**^**)**						
Digestible L (DL) intake	197^a^	231^b^	239^bc^	260^c^	4.7	<0.001
L gain	104^a^	211^b^	232^bc^	255^c^	7.6	<0.001
L retention efficiency (% DL)	53.1^a^	91.3^b^	94.9^b^	96.0^b^	3.25	<0.001
Fecal L losses	33.6^b^	20.5^a^	16.3^a^	16.9^a^	2.20	0.002
Non-fecal L losses	92.4^b^	20.2^a^	12.1^a^	10.6^a^	6.90	<0.001
**Energy (E) balance (kJ kg ABW**^**−1**^ **day**^**−1**^**)**						
Digestible E (DE) intake	259^a^	298^b^	308^bc^	336^c^	7.2	<0.001
Metabolizable E	238^a^	276^b^	286^bc^	311^c^	6.8	<0.001
E gain	84.5^a^	129^b^	136^b^	143^b^	4.0	<0.001
E retention efficiency (% DE)	32.7^a^	43.3^b^	44.1^b^	42.7^b^	1.26	<0.001
Fecal E losses	65.4	60.3	54.5	56.4	2.55	0.067
Non-fecal E losses	20.2^a^	22.5^bc^	22.4^ab^	24.7^c^	0.50	0.002
Total heat loss	154	147	150	168	5.7	0.118

Algae0, commercial-based diet without algae blend inclusion (control diet); Algae2, control diet with 2% algae blend inclusion; Algae4, control diet with 4% algae blend inclusion; Algae6, control diet with 6% algae blend inclusion; SEM, standard error of the mean; ABW, average body weight.

a−c Means in the same line with different superscripts are statistically different (*p* < 0.05).

### 3.3. Growth performance and feed utilization

Most growth performance and feed utilization parameters were affected by algae blend feeding (*p* < 0.05; Table 4). Final fish BW and length were improved, with the BW of fish fed Algae6 being 70% higher than that of fish fed Algae0. Algae blend supplementation promoted DGI, VSI, and HIS compared to the control (0%), but no differences were observed among inclusion levels. The lowest FCR was observed with 2% and 4% of algae blend and the highest with Algae0. The PER was the highest with Algae4, not differing from Algae2 and Algae 6. The algae blend also improved the condition factor, which was highest at the 4% and 6% inclusion levels.

**Table 4:** Growth performance and feed utilization of European seabass juveniles fed the experimental diets.

	**Diet**				**SEM**	***p-*** **value**
	**Algae0**	**Algae2**	**Algae4**	**Algae6**		
**Growth**						
Final body weight (g)	37.3^a^	54.4^b^	56.6^b^	62.9^c^	1.35	<0.001
Final length (cm)	15.6^a^	17.1^b^	17.1^b^	17.7^c^	0.14	<0.001
Specific growth rate	1.23^a^	1.62^b^	1.65^b^	1.77^b^	0.009	<0.001
Feed conversion ratio	1.35^b^	1.24^a^	1.22^a^	1.26^ab^	0.025	0.021
Protein efficiency ratio	1.41^a^	1.53^ab^	1.56^b^	1.50^ab^	0.028	0.024
Condition factor	0.96^a^	1.07^b^	1.11^c^	1.12^c^	0.009	<0.001
Daily growth index	1.13^a^	1.59^b^	1.64^b^	1.79^b^	0.060	<0.001
**Somatic indices (%)**						
Viscerosomatic index	6.14^a^	7.53^b^	7.88^b^	8.38^b^	0.245	<0.001
Hepatosomatic index	0.953^a^	1.31^b^	1.33^b^	1.42^b^	0.036	<0.001
**Voluntary feed intake (g kg ABW**^**−1**^ **day**^**−1**^**)**						
Dry matter	14.9^a^	16.7^b^	16.8^b^	18.0^b^	0.32	0.001
Crude protein	7.82^a^	8.81^b^	8.80^b^	9.53^b^	0.170	0.001
Ether extract	2.30^a^	2.52^ab^	2.56^bc^	2.77^c^	0.049	0.001
Gross energy (kJ kg ABW^−1^ day^−1^)	324^a^	359^b^	363^bc^	392^c^	7.0	0.001
Survival (%)	100	99.3	98.6	100	0.52	0.219

Algae0, commercial-based diet without algae blend inclusion (control diet); Algae2, control diet with 2% algae blend inclusion; Algae4, control diet with 4% algae blend inclusion; Algae6, control diet with 6% algae blend inclusion; SEM, standard error of the mean; ABW, average body weight.

a − c Means in the same line with different superscripts are statistically different (*p* < 0.05).

Feed intake of DM and CP was promoted in diets with algae supplementation, with no differences among inclusion levels. Feed intake of EE and GE was the highest at 6% and 4% algae inclusion, the latter not differing from 2% (Table 4).

The survival rate was not affected by algae blend supplementation (*p* > 0.05; Table 4).

### 3.4. Histomorphology of anterior intestine

**Figure 1:**
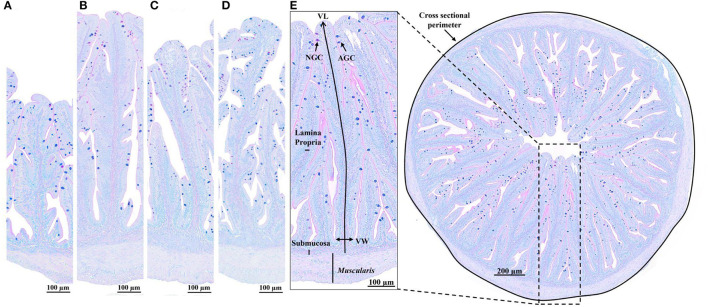
Histological sections (Alcian blue/PAS staining, pH = 2.5) of the anterior intestine of European seabass fed **(A)** commercial-based diet without algae blend inclusion (control diet; Algae0), **(B)** control diet with 2% algae blend inclusion (Algae2), **(C)** control diet with 4% algae blend inclusion (Algae4), and **(D)** control diet with 6% algae blend inclusion (Algae6). **(E)** Measured parameters: cross-sectional perimeter, villus length (VL), villus width (VW), muscularis, submucosa and lamina propria width, acid goblet cells (AGC, blue), and neutral goblet cells (NGC, pink).

The overall integrity of the anterior intestine was well preserved in all fish, but algae blend supplementation had a significant impact on its morphology (*p* < 0.05; Figure 1, Table 5).

**Table 5:** Anterior intestine morphology of European seabass juveniles fed the experimental diets.

	**Diet**				**SEM**	***p-*** **value**
	**Algae0**	**Algae2**	**Algae4**	**Algae6**		
**Quantitative analysis**						
Cross-sectional perimeter (mm)	7.70^a^	8.87^ab^	8.97^b^	9.77^b^	0.311	<0.001
Muscularis thickness (μm)	74.6	92.9	98.6	96.7	7.76	0.131
Submucosa width (μm)	21.9^a^	25.6^a^	32.3^b^	30.9^b^	1.37	<0.001
Lamina propria width (μm)	17.4^a^	21.3^ab^	22.5^b^	20.0^ab^	1.28	0.046
Absorption area (mm^2^)	2.16^a^	2.69^ab^	2.79^ab^	3.13^b^	0.228	0.038
Villus length (μm)	774^a^	927^ab^	966^b^	1033^b^	44.7	0.002
Villus width (μm)	104^a^	124^b^	132^b^	130^b^	5.2	0.002
Goblet cells number	42.9^a^	59.5^b^	56.4^ab^	62.2^b^	3.98	0.009
Neutral goblet cells	4.46	10.2	9.97	10.8	1.891	0.082
Acid goblet cells	38.5	49.3	46.4	51.4	4.48	0.209
Goblet cells per villus area (n° mm^2^^−1^)	20.4	23.6	20.6	20.3	1.74	0.487
Neutral goblet cells	2.07	4.37	3.62	3.89	0.928	0.343
Acid goblet cells	18.3	19.2	17.0	16.4	1.65	0.616
**Semi-quantitative analysis**						
Submucosa cellularity	1.28	1.44	1.33	1.56	0.172	0.675
Lamina propria cellularity	2.44	2.39	2.50	2.56	0.115	0.763
Mucosal folds	1.67	1.78	1.44	1.61	0.214	0.739
Inflammatory infiltrates	2.50	2.50	2.50	2.67	0.125	0.723
Enterocytes nucleolus position	2.11	1.83	1.94	1.78	0.178	0.571

Algae0, commercial-based diet without algae blend inclusion (control diet); Algae2, control diet with 2% algae blend inclusion; Algae4, control diet with 4% algae blend inclusion; Algae6, control diet with 6% algae blend inclusion; SEM, standard error of the mean.

a, b Means in the same line with different superscripts are statistically different (*p* < 0.05).

Most quantitative parameters were affected by the dietary inclusion of algae (*p* < 0.05), except for muscularis thickness, number of acid goblet cells, and number of goblet cells per villus area (*p* > 0.05). The cross-sectional perimeter, the submucosa width, and the villus length increased with algae inclusion levels, being the highest in fish fed Algae4 and Algae6 and the lowest in fish fed Algae0 and Algae2 for submucosa width; Algae2 was similar to other inclusion levels for cross-sectional perimeter and villus length. Compared to Algae0, lamina propria width was the highest in fish fed Algae4 while the highest absorption area was observed in fish fed Algae6. Goblet cell counts were the highest in fish fed Algae2 and Algae6.

Submucosal and lamina propria cellularity, mucosal folds, inflammatory infiltrates, and enterocyte nucleolus position ranged from normal to slightly altered tissue, not being significantly affected by algae blend inclusion (*p* > 0.05).

### 3.5. Whole-body composition

After 12 weeks of feeding, whole-body moisture and EE gradually decreased and increased, respectively, with the inclusion levels of the algae blend (*p* < 0.05; Table 6). Whole-body GE content of fish fed algae-supplemented diets was enhanced; no differences were observed among inclusion levels (*p* < 0.05). Ash and CP contents were not affected (*p* > 0.05).

**Table 6:** Whole-body composition (g 100 g^−1^, wet weight) of European seabass juveniles fed the experimental diets.

	**Diet**				**SEM**	***p-*** **value**
	**Algae0**	**Algae2**	**Algae4**	**Algae6**		
Moisture	69.0^c^	64.0^b^	62.8^ab^	62.3^a^	0.29	<0.001
Ash	4.67	4.35	4.39	4.13	0.225	0.457
Crude protein	18.3	18.1	18.6	18.4	0.14	0.239
Ether extract	8.89^a^	14.0^b^	15.0^bc^	15.9^c^	0.335	<0.001
Gross energy (kJ g^−1^)	7.42^a^	9.01^b^	9.30^b^	9.42^b^	0.143	<0.001

Algae0, commercial-based diet without algae blend inclusion (control diet); Algae2, control diet with 2% algae blend inclusion; Algae4, control diet with 4% algae blend inclusion; Algae6, control diet with 6% algae blend inclusion; SEM, standard error of the mean.

a−c Means in the same line with different superscripts are statistically different (*p* < 0.05).

Initial whole-body composition (g 100 g^−1^, wet weight): moisture: 70.5; ash: 4.85; crude protein: 16.6; ether extract: 7.54; gross energy: 6.81 (kJ g^−1^).

### 3.6. Muscle composition

Muscle moisture content decreased whereas total lipids and fatty acids content increased with algae supplementation compared to control (*p* < 0.05; Table 7), with no differences observed among levels. Algae supplementation did not affect muscle CP content (*p* > 0.05).

**Table 7:** Muscle nutritional value (g 100 g^−1^ wet weight, ww), fatty acids profile (g 100 g^−1^ total fatty acids), and lipid quality indices of European seabass juveniles fed the experimental diets.

	**Diet**				**SEM**	***p-*** **value**
	**Algae0**	**Algae2**	**Algae4**	**Algae6**		
**Nutritional value**						
Moisture	75.6^|b^	74.1^a^	73.5^a^	72.9^a^	0.30	<0.001
Crude protein	19.4	19.5	19.5	19.5	0.16	0.918
Total lipids	2.34^a^	3.32^b^	3.62^b^	4.08^b^	0.227	<0.001
Total fatty acids	2.26^a^	3.22^b^	3.26^b^	3.78^b^	0.169	<0.001
**Fatty acids**						
**Saturated fatty acids**						
Total SFA^1, 2, 3^	32.4	32.6	32.8	32.9	0.21	0.442
**Even-chain fatty acids**						
C14:0	3.64^a^	4.12^b^	4.49^c^	4.72^d^	0.052	<0.001
C16:0	21.7	21.7	21.7	21.7	0.12	0.990
C18:0	5.08^c^	4.81^bc^	4.53^ab^	4.30^a^	0.108	<0.001
C20:0	0.177^a^	0.187^b^	0.200^c^	0.213^d^	0.0020	<0.001
Sum^1^	30.9	31.1	31.2	31.2	0.20	0.751
**Odd-chain fatty acids**						
C15:0	0.402	0.416	0.426	0.438	0.0090	0.062
C17:0	0.442	0.434	0.441	0.465	0.0140	0.437
Sum^2^	0.86	0.869	0.886	0.924	0.0230	0.221
**Branched-chain fatty acids**						
*iso*-C17:0	0.302^a^	0.318^ab^	0.339^bc^	0.357^c^	0.0090	0.001
Sum^3^	0.620^a^	0.666^ab^	0.705^bc^	0.746^c^	0.0180	<0.001
**Monounsaturated fatty acids**						
C16:1 *n*-7	6.09^a^	6.75^b^	7.26^c^	7.71^d^	0.089	<0.001
C16:1 *n*-9	0.577	0.565	0.560	0.550	0.0130	0.552
C18:1 *n*-7	3.24	3.26	3.30	3.42	0.074	0.356
C18:1 *n*-9	20.3^a^	20.8^ab^	21.2^ab^	21.8^b^	0.34	0.027
C20:1 *n*-9	1.97^c^	1.91^bc^	1.84^ab^	1.77^a^	0.022	<0.001
C20:1 *n*-11	0.336^b^	0.319^ab^	0.311^a^	0.306^a^	0.0060	0.006
C22:1 *n*-9	0.255	0.249	0.248	0.248	0.0080	0.892
C22:1 *n*-11	1.48	1.43	1.38	1.33	0.042	0.076
C24:1 *n*-9	0.487	0.441	0.450	0.460	0.0170	0.271
Total MUFA^4^	34.9^a^	36.0^ab^	36.8^bc^	37.8^c^	0.41	<0.001
**Polyunsaturated fatty acids**						
C16:2 *n*-4	0.348^a^	0.408^b^	0.421^b^	0.448^c^	0.0060	<0.001
C16:3 *n*-4	0.260^a^	0.296^b^	0.323^bc^	0.355^c^	0.0090	<0.001
C16:4 *n*-1	0.485^a^	0.515^b^	0.530^b^	0.555^c^	0.0060	<0.001
C18:2 *n*-6	5.36^d^	4.81^c^	4.41^b^	4.04^a^	0.057	<0.001
C18:3 *n*-3	0.797^c^	0.777^b^	0.767^ab^	0.757^a^	0.0050	<0.001
C18:3 *n*-6	0.211^c^	0.184^b^	0.150^a^	0.136^a^	0.0040	<0.001
C18:4 *n*-3	1.06^a^	1.12^a^	1.21^b^	1.30^c^	0.020	<0.001
C20:2 *n*-6	0.376^c^	0.349^b^	0.321^a^	0.318^a^	0.0060	<0.001
C20:4 *n*-3	0.410^a^	0.416^ab^	0.433^bc^	0.445^c^	0.0050	<0.001
C20:4 *n*-6	0.941^b^	0.807^a^	0.758^a^	0.684^a^	0.0320	<0.001
C20:5 *n*-3 (EPA)	9.09	9.05	9.22	9.33	0.136	0.433
C21:5 *n*-3	0.300^c^	0.267^bc^	0.236^ab^	0.216^a^	0.0110	<0.001
C22:5 *n*-3	1.26^c^	1.23^bc^	1.15^ab^	1.07^a^	0.028	<0.001
C22:6 *n*-3 (DHA)	11.43^d^	10.8^c^	10.1^b^	9.31^a^	0.121	<0.001
Sum PUFA *n*-3^5^	24.4^c^	23.7^bc^	23.2^ab^	22.5^a^	0.25	<0.001
Sum PUFA *n*-6^6^	7.11^d^	6.36^c^	5.84^b^	5.37^a^	0.077	<0.001
Total PUFA^5, 6, 7^	32.7^c^	31.4^b^	30.4^ab^	29.3^a^	0.31	<0.001
**Ratios**						
PUFA/SFA	1.01^c^	0.961^b^	0.928^ab^	0.891^a^	0.0100	<0.001
*n*-6/*n*-3	0.291^d^	0.268^c^	0.251^b^	0.239^a^	0.0030	<0.001
EPA+DHA (mg 100 g^−1^ ww)	441^a^	611^b^	602^b^	674^b^	0318	<0.001
**Lipid quality indices**						
AI	0.577^a^	0.611^b^	0.636^c^	0.652^d^	0.0040	<0.001
TI	0.295^a^	0.314^b^	0.316^b^	0.325^b^	0.0030	<0.001
h/H ratio	1.94^c^	1.87^b^	1.82^ab^	1.78^a^	0.015	<0.001
Flesh quality score	20.5^c^	19.8^bc^	19.4^ab^	18.6^a^	0.22	<0.001

Algae0, commercial-based diet without algae blend inclusion (control diet); Algae2, control diet with 2% algae blend inclusion; Algae4, control diet with 4% algae blend inclusion; Algae6, control diet with 6% algae blend inclusion; SEM, standard error of the mean; SFA, saturated fatty acids; MUFA, monounsaturated fatty acids; PUFA, polyunsaturated fatty acids; *n*-6/*n*-3 ratio, *n*-6 PUFA to sum of *n*-3 PUFA ratio; EPA, eicosapentaenoic acid; DHA, docosahexaenoic acid; AI, atherogenicity index; TI, thrombogenicity index; h/H ratio, hypocholesterolemic to hypercholesterolemic fatty acids ratio.

^1^Sum includes C10:0, C12:0, C22:0, and C24:0.

^2^Sum includes C13:0.

^3^Sum includes *iso*-C14:0, *iso*-C15:0, *anteiso*-C15:0, *iso*-C16:0, and *anteiso*-C17:0.

^4^Sum includes C14:1 *n*-5, C17:1 *n*-7, and C20:1 *n*-7.

^5^Sum includes 20:3 *n*-3.

^6^Sum includes C20:3 *n*-6, C22:2 *n*-6, and C22:4 *n*-6.

^7^Sum includes 18:3 *n*-4.

a−d Means in the same line with different superscripts are statistically different (*p* < 0.05).

The muscle fatty acids profile was greatly affected by algae blend supplementation (*p* < 0.05; Table 7). Total SFA remained unchanged, reflecting the effect on C16:0 (*p* > 0.05). Total MUFA, C16:1 *n*-7, and C18:1 *n*-9 proportions were the highest in fish fed with 6% algae blend and the lowest in those fed control (0%) diets. Conversely, C20:1 *n*-9 and C20:1 *n*-11 decreased with algae blend inclusion compared to Algae0. Dietary inclusion of the algae blend decreased the proportions of all individual *n*-6 PUFA, leading to a decrease in total *n*-6 PUFA by 24.5%. Effects on *n*-3 PUFA differed among individual fatty acids. Dietary inclusion of the algae blend increased C18:4 *n*-3 and C20:4 *n*-3 and decreased C21:5 *n*-3, C22:5 *n*-3, and DHA proportions in muscle. Muscle EPA remained unchanged (*p* > 0.05). Overall, the total *n*-3 PUFA proportion gradually decreased with algae supplementation, with Algae6 being 7.8% lower than Algae0. The observed decrease in PUFA *n*-6 and *n*-3 led to a decrease in total PUFA and *n*-6/*n*-3 ratio with algae blend inclusion levels.

Although the PUFA proportions decreased with the algae blend inclusion, the higher lipid and fatty acids content of algae blend-fed European seabass muscle led to an increase in the essential fatty acids (EPA + DHA) content by nearly 43%; no differences were found among inclusion levels.

Algae blend supplementation increased the thrombogenicity and the atherogenicity indices (*p* < 0.05; Table 7); no differences were observed among levels in the former, while a gradual increase with increasing algae inclusion levels was observed in the latter. The h/H ratio and flesh quality score decreased with algae inclusion levels (*p* < 0.05), in fish fed Algae0 being the highest and in those fed Algae6 being the lowest.

### 3.7. Color of skin and muscle, and texture of muscle

Dorsal skin color parameters were affected by algae blend feeding (*p* < 0.05; Table 8). Skin brightness (L^*^) was the highest in control fish and the lowest in those fed Algae2, with Algae4 and Algae6 not differing from Algae0 and Algae2. The algae blend reduced the greenness (a^*^) of the skin compared to the control, while Alage4 and Algae6 increased the yellowness (b^*^) compared to Algae2, although not differing from Algae0. Chroma (C^*^) was the highest in the skin of fish fed Algae4 and the lowest in those fed Algae2. The skin hue angle (h) of fish fed Algae4 and Algae6 was lower compared to those fed Algae0 and Algae2, reflecting a less greenish skin tone.

**Table 8:** Skin and muscle color and muscle texture of European seabass juveniles fed the experimental diets.

	**Diet**				**SEM**	***p-*** **value**
	**Algae0**	**Algae2**	**Algae4**	**Algae6**		
**Color**						
**Skin**						
L^*^	69.1^b^	63.4^a^	64.7^ab^	67.3^ab^	1.40	0.027
a^*^	−5.76^a^	−4.66^b^	−4.59^b^	−4.55^b^	0.144	<0.001
b^*^	9.67^ab^	9.07^a^	10.6^b^	10.4^b^	0.327	0.007
C^*^	11.3^ab^	10.2^a^	11.5^b^	11.3^ab^	0.31	0.020
h	121^b^	118^b^	114^a^	114^a^	0.9	<0.001
**Muscle**						
L^*^	51.4	52.5	51.7	50.9	0.92	0.637
a^*^	−3.26	−3.60	−3.57	−3.29	0.161	0.308
b^*^	6.65^b^	5.54^a^	5.25^a^	5.70^ab^	0.260	0.002
C^*^	7.51^b^	6.72^a^	6.43^a^	6.67^a^	0.203	0.002
h	118^a^	123^ab^	125^b^	120^ab^	1.9	0.038
**Muscle texture**						
Hardness	1.46^b^	1.26^ab^	1.20^a^	1.06^a^	0.068	0.001
Adhesiveness	−0.111^a^	−0.0542^b^	−0.0645^b^	−0.0560^b^	0.00886	<0.001
Springiness	1.17	1.19	1.23	1.32	0.057	0.264
Cohesiveness	0.242	0.265	0.269	0.255	0.0148	0.573
Chewiness	0.426	0.377	0.403	0.336	0.0403	0.437
Resilience	0.596	0.738	0.654	0.904	0.0885	0.087

Algae0, commercial-based diet without algae blend inclusion (control diet); Algae2, control diet with 2% algae blend inclusion; Algae4, control diet with 4% algae blend inclusion; Algae6, control diet with 6% algae blend inclusion; SEM, standard error of the mean; L^*^, lightness degree; a^*^, redness/greeness degree; b^*^, yellowness/blueness degree; C^*^, chroma value; h, hue angle value.

a, b Means in the same line with different superscripts are statistically different (*p* < 0.05).

Muscle brightness (L^*^) and greenness (a^*^) were not affected by algae blend inclusion levels, while yellowness (b^*^) was the highest in fish fed Algae0 and the lowest in those fed Algae2 and Algae4 (Table 7); Algae6 fish did not differ from other diets. Compared to the control, algae blend inclusion reduced muscle chroma (C^*^), with no differences among inclusion levels. Conversely, the hue angle (h) was increased by algae feeding, with the highest value found in the muscle of fish fed Algae4 (more greenish) and the lowest in that of fish fed Algae0 (Table 8).

Regarding texture (Table 8), muscle hardness was the lowest in fish fed Algae4 and Algae6 and the highest in those fed the control diet (Algae0); Algae2 did not differ from the other levels. Muscle adhesiveness increased in fish fed algae blend, regardless of supplementation level. Muscle springiness, cohesiveness, and chewiness were not affected by algae supplementation. A trend toward increased muscle resilience with algae supplementation was observed.

## 4. Discussion

To ensure aquaculture sustainability, challenges related to aquafeed formulation and ingredient selection must be addressed, in line with the circular economy, the Blue Growth strategy of the European Union, and the Sustainable Development Goals of the 2030 Agenda. In recent years, algae have emerged as alternative aquafeed ingredients due to their nutritional and functional values and lower environmental footprint (13, 72), particularly macroalgae produced in integrated multi-trophic aquaculture (IMTA) systems and microalgae produced locally. Although dietary supplementation of micro- or macroalgae species as sustainable alternative aquafeeds to fishmeal and fish oil (13, 73, 74) or plant source ingredients (34, 75) has been assessed, the synergetic effects of the blend of micro- and macroalgae remain largely unexploited. The present study addressed this gap and unveiled the potential of dietary supplementation of a commercial blend of macro- (*Ulva* sp. and *G. gracilis*) and microalgae (*C. vulgaris* and *N. oceanica*) species up to 6% (DM basis) in digestibility, growth performance, and muscle nutritional value and quality of European seabass juveniles.

The functional potential of this algae blend has recently been suggested based on its chemical composition and bacteriostatic and bactericidal activities evidenced *in vitro* against some of the most common fish pathogenic bacteria (23). To the best of our knowledge, no other study has yet evaluated an algae blend composed of these four species in an *in vivo* study. The composition of macro- and microalgae is known to vary between species and within species with biotic and abiotic growth factors (76). Nonetheless, the overall proximate composition and amino acids content of the blend previously analyzed (23) are in broad agreement with the present results. The most relevant differences were in the polysaccharide content and the fatty acids content and profile. The commercial blend used in the present study had lower polysaccharides content (148 vs. 341 g kg^−1^) and total FA content (54.9 vs. 79.0 g kg^−1^), but higher PUFA *n*-3 (22.0 vs. 17.6% total FA) and EPA (10.8 vs. 6.91% total FA) proportions than that previously reported (23). The high content of macro (e.g., magnesium, potassium, and sodium) and trace elements (e.g., iron, manganese, and zinc) reported here further support the interest in the algae blend as functional aquafeed ingredients. However, the levels of toxic elements such as aluminum, arsenic, and copper may limit the inclusion level of the algae blend in fish diets.

In the present study, the commercial blend of *Ulva* sp., *G. gracilis, C. vulgaris*, and *N. oceanica* replaced protein-rich plant ingredients and had a positive effect if included up to 6% (DM basis): growth performance, feed intake, utilization efficiency, and body composition of *D. labrax* juveniles were enhanced compared to those fed the control diet (Algae0), a commercial-type formulated plant-based diet with moderate (125 g kg^−1^ DM basis) inclusion of fishmeal. These remarkable results were mostly unexpected as most studies evaluating dietary inclusion of mixtures of macroalgae species, microalgae species, or a combination of both have reported neutral to negative effects on growth performance and feed utilization in carnivorous fish. Indeed, the mixture of red macroalgae species (*Pyropia columbina* and *Gracilaria chilensis*) included up to 1.0% in diets for Atlantic salmon (77) and *Fucus* sp., *Gracilaria* sp., and *Ulva* sp. at 7.5% in European seabass (31, 52, 78) diets did not affect growth performance and feed intake. Similarly, no effect on growth performance and feed utilization was observed on meager fed 10% *Nannochloropsis gaditana, Tisochrysis lutea, Rhodomonas lens*, and *Isochrysis galbana* (79), and red sea bream fed 25% *Nannochloropsis* sp. and *Schizochytrium* sp. or 45% *Nannochloropsis* sp., *Chlorella* sp., and *Schizochytrium* sp. (80). On the other hand, the inclusion of *Nannochloropsis* sp. and *Isochrysis* sp. up to 11.9% reduced the feed intake and growth performance of Atlantic cod (81) and *Schizochytrium limacinum* and *N. oceanica* supplementation up to 17% reduced rainbow trout growth performance (82). The negative impact of microalgae blends was suggested to be due to their low palatability (81). The combination of macroalgae (*G. gracilis*) and microalgae (*N. oceanica*) species included at 30% did not affect European seabass growth performance or feed utilization (54).

Effects of dietary algae inclusion are species-specific with ideal inclusion levels varying with algae species and fish species (42, 72). In general, carnivorous fish, such as European seabass, digest algae recalcitrant cell walls more poorly than herbivorous fish due to the shorter intestine, the main organ for digestion and absorption (83). However, in the present study, DM, OM, CP, total lipids, GE, and most FA ADC were higher in diets with algae blend supplementation. These results contrast with the consistently reduced digestibility reported in the literature with algae inclusion, which has been attributed to algae cell walls complex polysaccharides that can resist enzymatic degradation in the stomach and small intestine of monogastric animals (84) and reduce the availability of intracellular nutrients (29, 54, 85, 86), namely ulvans in *Ulva* sp., carrageenans in *Gracilaria* sp. (16), cellulosic polymers and glucosamine, a chitin-like glycan, in *Chlorella* sp. (87), and algaenans (outer layer) and cellulosic polymers (inner layer) in *Nannochloropsis* sp. (88). We hypothesize that the ADC improvement observed with algae blend inclusion may be related to the plant-based reference diet used in this study. Experimental diets were formulated to include the algae blend at the expense of wheat gluten and whole peas, keeping constant the fishmeal and fish oil levels across diets. Whole peas were the main ingredient replaced by the algae blend (11% in Algae2, 24% in Algae4, and 32% in Algae 6). Peas (*Pisum sativum*) are moderate sources of protein (*c.a*. 22% DM basis) with low sulfur-containing amino acids, high polysaccharides, and low lipid content, but also contain antinutritional factors such as tannins, phytic acid, saponins, and trypsin inhibitor activities (89). Thus, we hypothesize that the lower digestibility observed in the control group (Algae0) with a higher pea content may be due to antinutritional factors, which may have affected the digestion and absorption of nutrients, and consequently growth performance and body composition of *D. labrax* juveniles. Gouveia and Davies (90) found that whole pea meal inclusion at 20% and 40% (DM basis) had no negative effect on palatability, feed intake, and growth performance of European seabass juveniles, but reduced carbohydrates and energy digestibility. The lower digestibility may result from the complex matrix of highly digestible non-structural polysaccharides (starch) and low digestible structural polysaccharides (fiber) of whole peas, which was suggested to limit the nutrient digestion and assimilation in rainbow trout (91). Moreover, the processing of whole peas, as peeling and extrusion, can reduce or even eliminate the antinutritional factors that may compromise feed intake and growth of fish (92).

The morphological structure of the intestine is considered a biomarker of the nutritional and physiological status, with changes related to altered nutrient digestibility (93). In the present study, the algae blend promoted villus length and width and anterior intestinal absorption area, which suggests an enhanced ability to absorb nutrients. This can at least partially explain the greater digestibility of algae-supplemented diets and consequent better feed utilization and growth of *D. labrax* juveniles. In contrast, previous studies reported no effects or even a reduction of intestinal area, or villus length and width in carnivorous fish fed diets supplemented with individual *Ulva* sp., *Gracilaria* sp., *Chlorella* sp., or *Nannochloropsis* sp. (41, 54, 94, 95), or their mixtures (54, 79, 80). Fish fed the control diet (Algae0) had the lowest number of goblet cells. These mucin-producing cells produce gel-like layers that protect epithelial mucosa, facilitate digesta transport, and protect against bacterial invasion (96). Two main subtypes of mucins are produced along the gastrointestinal tract, neutral, and acidic mucins; the former is related to digestive and absorptive processes (97) and the latter to protection against bacterial translocation (96). The tendency for neutral mucins to increase further support enhanced nutrient digestion and absorption in fish fed diets supplemented with algae blend compared to the control diet. Algae blend did not affect submucosa and lamina propria cellularity and inflammatory infiltrates, while increased submucosa and lamina propria width compared to fish fed with no algae. Further studies are needed to assess the algae blend impact on posterior intestine morphology that best relates to inflammatory processes and microbiota abundance and diversity.

Algae blend dietary inclusion had no negative impact on fish protein retention efficiency or whole-body and muscle protein content. Conversely, lipid and energy retention efficiency were promoted, which was reflected in a higher whole-body lipid and energy content of fish fed algae blend. The most marked effect of algae blend supplementation was observed on body lipid content, which gradually increased with algae inclusion levels compared to the control. Lipid metabolism, including whole body lipid deposition and partitioning pattern, of carnivorous fish has been shown to be related to dietary energy intake and affected by dietary protein sources (marine *vs*. vegetable) that regulate lipogenic enzyme expressions and activities (98–100). In our study, a general increase in lipid deposition was observed in a fish fed algae blend for 12 weeks, with increased body and muscle lipid content and HSI. These findings are in line with the observed improvement in the digestibility of algae blend-supplemented diets and suggest the absence of bioactive compounds with lipotropic activity in the algae blend, thus contrasting with the findings of Tulli et al. (101) and suggestion of the presence of algae bioactive compounds with lipotropic activity.

Fish is the most important source of *n*-3 LC-PUFA EPA and DHA in the human diet (102). Although European seabass, like other marine finfish species, has the enzymatic ability for endogenous LC-PUFA biosynthesis, the low activity of enzymes involved in the desaturation/elongation pathway hampers the production of EPA and DHA from the C18 fatty acid precursor (α-linolenic acid; C18:3 *n*-3) at rates that meet physiological demands (103, 104). Thus, marine fish depend on dietary supplies of EPA and DHA to fulfill their essential *n*-3 PUFA requirements (102). Algae, particularly microalgae, may constitute alternative sustainable sources of *n*-3 LC-PUFA, although marked differences in the fatty acids profile are found among and within algae species (105). In addition, we hypothesize that the bioactive compounds present in algae can modulate the lipid metabolism of fish fed algae-supplemented diets, and prevent dietary fatty acids oxidation, with a putative impact on fish nutritional value and consumers' health.

In the present study, the experimental diets were formulated to include graded levels of algae blend at a constant fish oil content, thus ensuring high levels (>30 g kg^−1^ DM basis) of EPA and DHA. Dietary algae blend inclusion was found to alter the dorsal muscle fatty acids profile, mainly by promoting MUFA and reducing PUFA proportion while total SFA proportion remained unaffected. As the fatty acids profile and content in European seabass muscle have been reported to reflect dietary fatty acids (106–108), a stepwise increase in α-linolenic (C18:3 *n*-3), stearidonic (C18:4 *n*-3), arachidonic (C20:4 *n*-6), EPA, docosapentaenoic (C22:5 *n*-3) acids, and total *n*-3 PUFA and a decrease in linoleic acid (C18:2 *n*-6), DHA, total *n*-6 PUFA, and *n*-6/*n*-3 ratio were expected in the muscle mirroring the diet fatty acids profile. However, only linoleic acid, DHA, and total PUFA *n*-6 proportion and *n*-6/*n*-3 ratio followed the expected pattern, while most individual *n*-3 PUFA and all *n*-6 PUFA proportion decreased in the dorsal muscle. These results may suggest a potential for the algae blend to modulate the lipid metabolism of *D. labrax* juveniles through selective retention or catabolism of specific fatty acids. The observed decrease in linoleic and α-linolenic acids proportions in the muscle of fish fed algae supplemented diets is in agreement with previous reports in microalgae-supplemented diets for rainbow trout (82) and turbot (33), suggesting that these C18 PUFA may have been selectively catabolized. On the other hand, dietary algae blend supplementation up to 6% had no negative effect on muscle EPA, which may suggest a preferential deposition and retention of this essential fatty acid in the muscle of European seabass juveniles. The concomitant effect of the algae blend on muscle EPA and DHA proportion contrasts with previous studies that reported DHA selective deposition and retention in the flesh of marine fish species, including European seabass (51, 107), as a result of the high specificity transferases and low catabolism of DHA, whereas EPA is often selectively catabolized by β-oxidation (82, 107). The observed modifications in the muscle fatty acids profile may be due to the high PUFA content of experimental diets provided by fish oil and algae lipids, which may have partially suppressed *de novo* fatty acid synthesis (109) and thus affected the lipid metabolism of juvenile *D. labrax*. Further studies focused on intermediary metabolism are needed to clarify this point. Of particular importance is that the algae blend improved overall muscle fatty acid retention in these fish, resulting in an increased EPA and DHA (EPA+DHA) content (mg g^−1^ wet weight) to values well above the recommended 500 mg EPA+DHA per day to prevent coronary heart disease (110). Consumers would have to ingest 113 g of Algae0 fed or only 74.2 g of Algae6 fed European seabass filets. Seabass muscle lipid quality indices provide additional information on the effects of dietary algae blends on the flesh's nutritional and functional value. Atherogenicity and thrombogenicity indices are related to the risk of atherosclerosis and thrombosis, and the h/H ratio to cholesterol metabolism; lower indices and higher ratio relating to coronary health promotion (71, 111). Although dietary algae blend supplementation promoted AI and TI and reduced h/H ratio of seabass muscle, all values obtained were within the range considered to exert potential cardiovascular promoting effects (71, 111, 112). But a longer-term study should be carried out until the fish reach a commercial size to confirm the full potential of the algal blend in aquafeeds.

The skin color of the fish is of utmost importance for consumer acceptance (113). European seabass is appreciated by consumers for its white flesh, mild flavor, and low-fat content (114). In the present study, dietary algae blend supplementation altered the skin pigmentation of juvenile European seabass to a darker and less greenish color than observed in fish fed Algae0. While significant, the changes in skin pigmentation were small and mostly imperceptible to the naked eye. However, our results contrast with a more greenish skin of European seabass fed *T. suecica* (101) and *Isochrysis* sp. (108), and with the absence of effects of *G. vermicullophyla*. and *N. oceanica* supplementation, individually or as a mixture (54). In the present study, the dorsal filet color of fish fed control diet was more yellowish than those fed algae blend diets. This result agrees with the observation of Grigorakis (112) that a higher lipid content promotes a whiter color, as the muscle lipid content increased by 41.9%, 54.7%, and 66.0% with 2%, 4%, and 6% algae blend inclusion, respectively. The less yellowish color observed here may suggest an enhanced acceptance of European seabass juveniles fed up to 6% algae blend inclusion by consumers. However, these results were obtained in juvenile fish, and further studies are needed to assess the effects on pigmentation of commercial-sized fish and on consumers preference.

The fish texture is an important attribute for assessing quality, freshness, and palatability (115). A firmer texture is preferred for consumers and industry as it is considered an indicator of freshness (116), and soft filets pose difficulties to the fish processing industry (117). Algae blend supplementation reduced muscle hardness and improved adhesiveness of European seabass juveniles, compared to fish fed Algae0. These results suggest that the algae blend diminished the texture of seabass muscle, by presenting softer traits. The softer texture of algae-fed seabass muscle was associated with lower moisture and higher lipid content. The intramuscular lipid content is considered to enhance the fish flavor and provide a smoother, juicier mouthfeel, thereby improving muscle juiciness (112). Improved juiciness may counteract the softer texture of algae blend-fed European sea juveniles. Thus, longer feeding trials and a sensory panel evaluation should be carried out to fully assess the impact of algae blend supplementation on texture traits.

## 5. Conclusion

Supplementation of *Ulva* sp., *G. gracilis, C. vulgaris*, and *N. oceanica* blend up to 6% to a commercial-type plant-based diet significantly improved the digestibility and feed utilization of diets as well as anterior intestine absorption area, feed intake, and growth performance of European seabass juveniles. Muscle nutritional value and quality were also improved by algae blend supplementation. Of particular importance is the increase in essential fatty acids (EPA+DHA) content, which allows for achieving daily intake recommendations for EPA and DHA even with lower consumption of fish.

## Data availability statement

The original contributions presented in the study are included in the article/Supplementary material, further inquiries can be directed to the corresponding author.

## Ethics statement

The animal study was reviewed and approved by Animal Welfare and Ethics Body of CIIMAR (ORBEA-CIIMAR 06-2016) and licensed by the Portuguese Veterinary Authority (1005/92, DGAV-Portugal).

## Author contributions

MM, LV, AF, HA, and JS conceived and designed the study. CM, OP, TS, and MF conducted the research and performed the analyses. CD-M, AC, AA, AF, LV, and MM participated in the study coordination. CM and MM drafted the manuscript. All authors contributed to the manuscript revision, and read and approved the submitted version.
