# Supplementary material for: A commercial blend of macroalgae and microalgae promotes digestibility, growth performance, and muscle nutritional value of European seabass (Dicentrarchus labrax L.) juveniles
Source: Front Nutr. 2023 Apr 17;10:1165343. doi: 10.3389/fnut.2023.1165343 (PMC10150028; doi:10.3389/fnut.2023.1165343)
Supplement: Supplementary file 1 [file Table_1.docx]

Supplementary Material

A commercial blend of macroalgae and microalgae promotes digestibility, growth performance, and muscle nutritional value of European seabass (*Dicentrarchus labrax* L.) juveniles

Cátia S. C. Mota, Olívia Pinto, Tiago Sá, Mariana Ferreira, Cristina Delerue-Matos, Ana R. J. Cabrita, Agostinho Almeida, Helena Abreu, Joana Silva, António J. M. Fonseca, Luísa M. P. Valente, Margarida R. G. Maia^*^

*** Correspondence:** Margarida R.G. Maia: [mrmaia@icbas.up.pt](mailto:mrmaia@icbas.up.pt)

**Table S1.** Detailed fatty acids, mineral and non-essential amino acids composition (g kg^-1^ dry matter) of the algae blend and experimental diets.

|  | **Algae blend** | **Diet** | | | |
| --- | --- | --- | --- | --- | --- |
|  |  | **Algae0** | **Algae2** | **Algae4** | **Algae6** |
| **Fatty acids** |  |  |  |  |  |
| Total fatty acids | 54.8 | 159 | 161 | 162 | 162 |
| **Saturated fatty acids** |  |  |  |  |  |
| Total SFA | 19.4 | 52.8 | 52.5 | 52.6 | 52.2 |
| **Even-chain fatty acids** |  |  |  |  |  |
| C12:0 | 0.176 | 0.231 | 0.200 | 0.209 | 0.185 |
| C14:0 | 2.39 | 10.6 | 10.5 | 10.9 | 10.4 |
| C16:0 | 13.5 | 31.6 | 31.5 | 31.4 | 31.2 |
| C18:0 | 0.536 | 6.13 | 6.05 | 6.16 | 6.08 |
| C20:0 | 0.040 | 0.546 | 0.565 | 0.585 | 0.584 |
| C22:0 | 0.173 | 0.293 | 0.292 | 0.308 | 0.297 |
| C24:0 | 0.076 | 0.234 | 0.237 | 0.238 | 0.232 |
| Sum | 17.0 | 49.7 | 49.4 | 49.9 | 49.0 |
| **Odd-chain fatty acids** |  |  |  |  |  |
| C13:0 | 0.008 | 0.056 | 0.058 | 0.059 | 0.054 |
| C15:0 | 0.169 | 0.805 | 0.799 | 0.825 | 0.789 |
| C17:0 | 0.184 | 0.772 | 0.772 | 0.785 | 0.758 |
| Sum | 0.361 | 1.63 | 1.63 | 1.67 | 1.60 |
| **Branched-chain fatty acids** |  |  |  |  |  |
| *iso-*C14:0 | 0.029 | 0.059 | 0.072 | 0.077 | 0.067 |
| *iso*-C15:0 | 0.365 | 0.324 | 0.341 | 0.361 | 0.361 |
| *anteiso*-C15:0 | 0.110 | 0.096 | 0.089 | 0.092 | 0.090 |
| *iso-*C16:0 | 0.0117 | 0.120 | 0.119 | 0.126 | 0.121 |
| *iso-*C17:0 | 1.37 | 0.708 | 0.732 | 0.780 | 0.775 |
| *anteiso-*C17:0 | 0.036 | 0.116 | 0.110 | 0.116 | 0.105 |
| Sum | 2.03 | 1.42 | 1.46 | 1.55 | 1.52 |
| **Monounsaturated fatty acids** |  |  |  |  |  |
| C14:1 *n*-5 | 0.026 | 0.051 | 0.050 | 0.052 | 0.052 |
| C16:1 *n*-7 | 7.39 | 12.4 | 12.5 | 12.5 | 12.5 |
| C16:1 *n*-9 | 1.01 | 0.398 | 0.419 | 0.448 | 0.440 |
| C17:1 *n*-7 | 2.67 | 0.092 | 0.170 | 0.229 | 0.278 |
| C18:1 *n*-7 | 1.36 | 4.63 | 4.61 | 4.75 | 4.55 |
| C18:1 *n*-9 | 2.13 | 17.9 | 17.7 | 17.5 | 17.2 |
| C20:1 *n*-7 | 0.011 | 0.353 | 0.356 | 0.365 | 0.333 |
| C20:1 *n*-9 | 0.038 | 2.44 | 2.43 | 2.50 | 2.37 |
| C20:1 *n*-11 | 0.012 | 0.296 | 0.290 | 0.288 | 0.277 |
| C22:1 *n*-9 | 0.027 | 0.380 | 0.379 | 0.395 | 0.386 |
| C22:1 *n*-11 | 0.033 | 2.44 | 2.42 | 2.48 | 2.36 |
| C24:1 *n*-9 | 0.021 | 0.820 | 0.826 | 0.843 | 0.809 |
| Total MUFA | 14.7 | 42.2 | 42.2 | 42.4 | 41.5 |

**Table S1 (Continued)**

|  | **Algae blend** | **Diet** | | | |
| --- | --- | --- | --- | --- | --- |
|  |  | **Algae0** | **Algae2** | **Algae4** | **Algae6** |
| **Polyunsaturated fatty acids** |  |  |  |  |  |
| C16:2 *n*-4 | 0.089 | 1.40 | 1.42 | 1.48 | 1.42 |
| C16:3 *n*-4 | ND | 1.63 | 1.66 | 1.67 | 1.69 |
| C16:4 *n*-1 | 0.078 | 2.85 | 2.95 | 3.03 | 3.00 |
| C18:2 *n*-6 | 4.00 | 10.7 | 10.6 | 10.3 | 10.1 |
| C18:3 *n*-3 | 5.52 | 1.81 | 1.92 | 2.00 | 2.09 |
| C18:3 *n*-4 | 0.044 | 0.157 | 0.159 | 0.169 | 0.177 |
| C18:3 *n*-6 | 0.144 | 0.291 | 0.304 | 0.309 | 0.312 |
| C18:4 *n*-3 | 0.439 | 3.68 | 3.84 | 3.91 | 3.98 |
| C20:2 *n*-6 | 0.040 | 0.264 | 0.265 | 0.267 | 0.269 |
| C20:3 *n*-3 | 0.059 | 0.152 | 0.157 | 0.166 | 0.159 |
| C20:3 *n*-6 | 0.233 | 0.213 | 0.217 | 0.226 | 0.230 |
| C20:4 *n*-3 | 0.040 | 1.02 | 1.06 | 1.13 | 1.10 |
| C20:4 *n*-6 | 4.01 | 1.22 | 1.35 | 1.49 | 1.55 |
| C20:5 *n*-3 (EPA) | 5.93 | 21.1 | 22.3 | 23.1 | 23.6 |
| C21:5 *n*-3 | 0.014 | 0.370 | 0.380 | 0.396 | 0.404 |
| C22:2 *n*-6 | 0.002 | 0.018 | 0.023 | 0.017 | 0.029 |
| C22:4 *n*-6 | 0.013 | 0.099 | 0.118 | 0.120 | 0.119 |
| C22:5 *n*-3 | 0.055 | 2.20 | 2.31 | 2.39 | 2.42 |
| C22:6 *n*-3 (DHA) | ND | 15.3 | 15.2 | 15.3 | 15.2 |
| Sum PUFA *n*-3 | 12.1 | 45.6 | 47.2 | 48.4 | 49.0 |
| Sum PUFA *n*-6 | 8.4 | 12.8 | 12.9 | 12.7 | 12.6 |
| Total PUFA | 20.7 | 64.5 | 66.2 | 67.5 | 67.8 |
| *n*-6/*n*-3 ratio | 0.700 | 0.281 | 0.273 | 0.263 | 0.258 |
| **Minerals** |  |  |  |  |  |
| Total minerals | 112 | 28.8 | 30.8 | 33.4 | 35.9 |
| **Macro elements** |  |  |  |  |  |
| Calcium | 6.57 | 12.8 | 12.8 | 13.0 | 12.9 |
| Magnesium | 29.6 | 4.62 | 5.05 | 5.72 | 6.45 |
| Phosphorus | 12.4 | 13.6 | 13.7 | 13.9 | 14.3 |
| Potassium | 30.1 | 8.77 | 9.36 | 10.3 | 11.4 |
| Sodium | 43.1 | 2.07 | 3.00 | 3.82 | 4.52 |
| Total | 109 | 28.3 | 30.2 | 32.8 | 35.3 |
| **Trace elements (µg g^-1^ DM)** |  |  |  |  |  |
| Aluminum | 791 | 77.7 | 108 | 107 | 110 |
| Arsenic | 4.69 | 1.89 | 1.70 | 1.87 | 1.95 |
| Barium | 6.84 | 6.73 | 5.77 | 5.99 | 6.31 |
| Boron | 79.8 | 16.3 | 14.9 | 16.4 | 18.3 |
| Cadmium | 0.183 | 0.311 | 0.270 | 0.276 | 0.277 |
| Chromium | 7.47 | 3.72 | 3.29 | 3.30 | 3.52 |
| Cobalt | 0.826 | 0.124 | 0.136 | 0.154 | 0.178 |

**Table S1 (Continued)**

|  | **Algae blend** | **Diet** | | | |
| --- | --- | --- | --- | --- | --- |
|  |  | **Algae0** | **Algae2** | **Algae4** | **Algae6** |
| Copper | 26.9 | 12.2 | 12.5 | 11.2 | 11.5 |
| Iodine | 41.9 | 0.757 | 1.57 | 2.41 | 3.26 |
| Iron | 1610 | 220 | 259 | 290 | 314 |
| Lead | 1.06 | 0.137 | 0.159 | 0.126 | 0.168 |
| Lithium | 1.67 | 0.157 | 0.164 | 0.190 | 0.213 |
| Manganese | 147 | 27.7 | 29.0 | 30.3 | 32.8 |
| Molybdenum | 1.47 | 2.04 | 1.73 | 1.79 | 1.77 |
| Nickel | 4.79 | 1.38 | 1.25 | 1.00 | 1.13 |
| Rubidium | 9.90 | 14.0 | 12.3 | 12.5 | 12.7 |
| Selenium | 0.361 | 0.487 | 0.398 | 0.433 | 0.454 |
| Strontium | 65.9 | 19.6 | 17.8 | 18.7 | 20.2 |
| Tin | 0.247 | 0.091 | 0.178 | 0.101 | 0.099 |
| Vanadium | 3.90 | 3.36 | 2.95 | 3.00 | 3.19 |
| Zinc | 177 | 42.6 | 43.6 | 44.5 | 43.3 |
| Total | 2988 | 510 | 568 | 601 | 637 |
| **Amino acids** |  |  |  |  |  |
| Total amino acids | 358 | 524 | 528 | 524 | 529 |
| **Non-essential amino acids** |  |  |  |  |  |
| Alanine | 24.6 | 25.5 | 26.4 | 26.9 | 26.9 |
| Aspartic acid + Asparagine | 32.1 | 38.9 | 42.9 | 42.8 | 45.9 |
| Glutamic acid + Glutamine | 49.5 | 101 | 105 | 105 | 106 |
| Glycine | 24.7 | 32.9 | 31.6 | 31.9 | 37.0 |
| Proline | 21.8 | 36.8 | 38.4 | 38.4 | 37.0 |
| Serine | 18.2 | 27.9 | 28.8 | 29.0 | 26.4 |
| Total | 171 | 263 | 273.3 | 274 | 280 |

Algae0, commercial-based diet without algae blend inclusion (control diet); Algae2, control diet with 2% algae blend inclusion; Algae4, control diet with 4% algae blend inclusion; Algae6, control diet with 6% algae blend inclusion. SFA, saturated fatty acids; MUFA, monounsaturated fatty acids; PUFA, polyunsaturated fatty acids; *n*-6/*n*-3 ratio, *n*-6 PUFA to sum of *n*-3 PUFA ratio; EPA, eicosapentaenoic acid; DHA, docosahexaenoic acid; ND, not detected.

**Table S2.** Fatty acids apparent digestibility coefficients (ADC, %) of the experimental diets fed to European seabass juveniles.

|  | **Diet** | | | |  |  |
| --- | --- | --- | --- | --- | --- | --- |
|  | **Algae0** | **Algae2** | **Algae4** | **Algae6** | **SEM** | ***p-*value** |
| **Saturated fatty acids** |  |  |  |  |  |  |
| **Even-chain fatty acids** |  |  |  |  |  |  |
| C10:0 | 93.8 | 94.2 | 98.0 | 96.5 | 1.33 | 0.165 |
| C12:0 | 76.2 | 79.2 | 86.6 | 85.0 | 2.53 | 0.061 |
| C14:0 | 79.2^a^ | 88.2^b^ | 92.6^b^ | 92.4^b^ | 1.73 | 0.002 |
| C16:0 | 72.4^a^ | 82.5^b^ | 87.9^b^ | 88.0^b^ | 1.92 | 0.001 |
| C18:0 | 64.0^a^ | 75.4^b^ | 82.7^b^ | 83.9^b^ | 2.29 | <0.001 |
| C20:0 | 62.5^a^ | 76.0^b^ | 83.6^b^ | 84.5^b^ | 2.16 | <0.001 |
| C22:0 | 57.3^a^ | 65.9^ab^ | 74.4^bc^ | 75.0^c^ | 1.99 | <0.001 |
| C24:0 | 57.4^a^ | 66.2^b^ | 70.3^b^ | 71.7^b^ | 1.91 | 0.003 |
| **Odd-chain fatty acids** |  |  |  |  |  |  |
| C13:0 | 81.1^a^ | 89.6^ab^ | 94.4^b^ | 93.8^b^ | 1.95 | 0.005 |
| C15:0 | 74.9^a^ | 85.1^b^ | 90.0^b^ | 90.1^b^ | 1.88 | 0.001 |
| C17:0 | 68.1^a^ | 79.8^b^ | 85.8^b^ | 86.1^b^ | 2.21 | 0.001 |
| **Branched-chain fatty acids** |  |  |  |  |  |  |
| *iso*-C14:0 | 84.5^a^ | 92.5^b^ | 95.7^b^ | 95.2^b^ | 1.42 | 0.002 |
| *iso*-C15:0 | 79.8^a^ | 89.0^b^ | 92.3^b^ | 92.0^b^ | 1.75 | 0.003 |
| *anteiso*-C15:0 | 79.0^a^ | 90.0^b^ | 92.8^b^ | 91.2^b^ | 2.08 | 0.006 |
| *iso*-C16:0 | 76.8^a^ | 86.2^b^ | 90.7^b^ | 90.4^b^ | 1.83 | 0.002 |
| *iso*-C17:0 | 79.1^a^ | 87.8^b^ | 92.0^b^ | 91.8^b^ | 1.56 | 0.001 |
| *anteiso*-C17:0 | 73.3^a^ | 84.8^b^ | 90.0^b^ | 90.0^b^ | 2.26 | 0.002 |
| **Monounsaturated fatty acids** |  |  |  |  |  |  |
| C14:1 *n*-5 | 85.5^a^ | 93.1^b^ | 96.0^b^ | 95.3^b^ | 1.53 | 0.004 |
| C16:1 *n*-7 | 87.3^a^ | 93.0^b^ | 95.4^b^ | 95.2^b^ | 1.08 | 0.002 |
| C16:1 *n*-9 | 86.4 | 89.6 | 88.9 | 85.2 | 1.13 | 0.075 |
| C17:1 *n*-7 | 90.7 | 89.4 | 89.1 | 87.7 | 1.04 | 0.318 |
| C18:1 *n*-7 | 83.0^a^ | 90.3^b^ | 93.8^b^ | 93.9^b^ | 1.33 | 0.001 |
| C18:1 *n*-9 | 86.1^a^ | 91.5^b^ | 94.5^b^ | 94.8^b^ | 1.02 | 0.001 |
| C20:1 *n*-7 | 80.9^a^ | 88.9^b^ | 92.6^b^ | 92.4^b^ | 1.44 | 0.001 |
| C20:1 *n*-9 | 83.0^a^ | 90.6^b^ | 94.1^b^ | 94.3^b^ | 1.32 | <0.001 |
| C20:1 *n*-11 | 67.2^a^ | 88.0^ab^ | 93.9^b^ | 94.1^b^ | 4.67 | 0.011 |
| C22:1 *n*-9 | 78.5^a^ | 87.7^b^ | 92.3^b^ | 92.9^b^ | 1.69 | 0.001 |
| C22:1 *n*-11 | 81.3^a^ | 89.9^b^ | 93.9^b^ | 94.1^b^ | 1.52 | 0.001 |
| C24:1 *n*-9 | 63.6^a^ | 76.4^b^ | 82.3^b^ | 83.4^b^ | 2.26 | <0.001 |
| **Polyunsaturated fatty acids** |  |  |  |  |  |  |
| C16:2 *n*-4 | 94.3^a^ | 95.8^ab^ | 97.2^b^ | 97.2^b^ | 0.38 | 0.002 |
| C16:3 *n*-4 | 96.6^a^ | 97.3^ab^ | 98.1^b^ | 98.1^b^ | 0.28 | 0.014 |
| C16:4 *n*-1 | 95.7 | 96.7 | 97.6 | 97.6 | 0.26 | 0.003 |
| C18:2 *n*-6 | 90.4^a^ | 90.5^a^ | 93.5^b^ | 93.4^b^ | 0.39 | <0.001 |
| C18:3 *n*-3 | 90.6^ab^ | 90.0^a^ | 92.7^c^ | 92.2^bc^ | 0.38 | 0.003 |

**Table S2 (Continued)**

|  | **Diet** | | | |  |  |
| --- | --- | --- | --- | --- | --- | --- |
|  | **Algae0** | **Algae2** | **Algae4** | **Algae6** | **SEM** | ***p-*value** |
| C18:3 *n*-4 | 91.4^a^ | 91.8^a^ | 93.6^b^ | 92.2^ab^ | 0.39 | 0.016 |
| C18:3 *n*-6 | 94.9^a^ | 95.9^ab^ | 97.2^c^ | 96.7^bc^ | 0.25 | <0.001 |
| C18:4 *n*-3 | 96.6^a^ | 97.2^ab^ | 98.0^b^ | 98.0^b^ | 0.27 | 0.015 |
| C20:2 *n*-6 | 87.9^a^ | 90.6^a^ | 93.5^b^ | 93.6^b^ | 0.62 | <0.001 |
| C20:3 *n*-3 | 89.9^a^ | 91.7^ab^ | 93.7^b^ | 93.4^b^ | 0.72 | 0.020 |
| C20:3 *n*-6 | 94.7 | 94.2 | 95.6 | 95.2 | 0.54 | 0.325 |
| C20:4 *n*-3 | 95.5^a^ | 96.1^ab^ | 97.2^b^ | 97.2^b^ | 0.37 | 0.028 |
| C20:4 *n*-6 | 95.1^b^ | 94.4^ab^ | 94.3^ab^ | 93.4^a^ | 0.36 | 0.047 |
| C20:5 *n*-3 (EPA) | 96.5 | 97.0 | 97.6 | 97.5 | 0.27 | 0.090 |
| C21:5 *n*-3 | 94.4^a^ | 95.2^ab^ | 96.5^b^ | 96.7^b^ | 0.43 | 0.015 |
| C22:4 *n*-6 | 97.0 | 96.8 | 97.3 | 96.9 | 0.21 | 0.457 |
| C22:5 *n*-3 | 95.4^a^ | 96.1^ab^ | 97.0^ab^ | 97.2^b^ | 0.36 | 0.030 |
| C22:6 *n*-3 (DHA) | 94.7^a^ | 95.7^ab^ | 96.7^b^ | 96.8^b^ | 0.35 | 0.008 |

Algae0, commercial-based diet without algae blend inclusion (control diet); Algae2, control diet with 2% algae blend inclusion; Algae4, control diet with 4% algae blend inclusion; Algae6, control diet with 6% algae blend inclusion; SEM, standard error of the mean; SFA, saturated fatty acids; MUFA, monounsaturated fatty acids; PUFA, polyunsaturated fatty acids; EPA, eicosapentaenoic acid; DHA, docosahexaenoic acid.

^a−c^Means in the same line with different superscripts are statistically different (*p* < 0.05).
